# SIRT3-PINK1-PKM2 axis prevents osteoarthritis via mitochondrial renewal and metabolic switch

**DOI:** 10.1038/s41413-025-00413-4

**Published:** 2025-03-14

**Authors:** Yaoge Deng, Mingzhuang Hou, Yubin Wu, Yang Liu, Xiaowei Xia, Chenqi Yu, Jianfeng Yu, Huilin Yang, Yijian Zhang, Xuesong Zhu

**Affiliations:** 1 Department of Orthopaedics, The First Affiliated Hospital of Soochow University, Soochow University, Suzhou, 215006 China; 2 Orthopaedic Institute, Medical College, Soochow University, Suzhou, 215000 China

**Keywords:** Bone, Pathogenesis

## Abstract

Maintaining mitochondrial homeostasis is critical for preserving chondrocyte physiological conditions and increasing resistance against osteoarthritis (OA). However, the underlying mechanisms governing mitochondrial self-renewal and energy production remain elusive. In this study, we demonstrated mitochondrial damage and aberrant mitophagy in OA chondrocytes. Genetically overexpressing PTEN-induced putative kinase 1 (PINK1) protects against cartilage degeneration by removing defective mitochondria. PINK1 knockout aggravated cartilage damage due to impaired mitophagy. SIRT3 directly deacetylated PINK1 to promote mitophagy and cartilage anabolism. Specifically, PINK1 phosphorylated PKM2 at the Ser127 site, preserving its active tetrameric form. This inhibited nuclear translocation and the interaction with β-catenin, resulting in a metabolic shift and increased energy production. Finally, a double-knockout mouse model demonstrated the role of the SIRT3-PINK1-PKM2 axis in safeguarding the structural integrity of articular joints and improving motor functions. Overall, this study provides a novel insight into the regulation of mitochondrial renewal and metabolic switches in OA.

## Introduction

Osteoarthritis (OA) is a prevalent disease that threatens human life and well-being by causing chronic pain and irreversible joint deformities.^1^ Chondrocytes are the only resident cells dispersed within cartilage lacunae, and their dysfunction can result in insufficient synthesis of cartilage extracellular matrix (C-ECM), contributing to OA development. At the subcellular level, mitochondria play crucial roles in maintaining cellular function and preventing cartilage degeneration.^2^ Mitochondrial charging holds great promise as a treatment for OA by enhancing energy production and restoring redox balance.^3^ However, in advanced stages of OA, it causes irreversible damage in a subset of impaired mitochondria, necessitating the activation of an intrinsic clearance and self-renewal mechanism. Selective mitochondrial autophagy, or mitophagy, is the process of selectively degrading damaged mitochondria.^4^ Literature reports a strong association between abnormal mitophagy and musculoskeletal disorders, such as bone loss^5^ and spinal cord injury.^6^ Although biomaterials have been developed to reactivate mitophagy in damaged chondrocytes and alleviate OA,^7^ the underlying mechanisms responsible for mitophagy in OA remain elusive. Mitophagy regulatory pathways are classified into two categories: the ubiquitin-dependent PTEN-induced putative kinase 1 (PINK1)–Parkin RBR E3 ubiquitin protein ligase (PRKN) pathway^8^ and the ubiquitin-independent receptor-mediated mitophagy involving BCL2 interacting protein 3 (BNIP3), BCL2 interacting protein 3 like (BNIP3L/NIX), and FUN14 domain containing 1 (FUNDC1).^9^ FUNDC1 activation via PFKP-mediated dephosphorylation protects cartilage by inducing mitophagy.^10^ However, excessive mitophagy caused by ECM stiffening may exert detrimental effects on chondrocyte health and drive OA pathogenesis.^11^ Consequently, intensive investigation into the dynamic role of mitophagy in cartilage degeneration is imperative.

Because cartilage is avascular, chondrocytes generate energy through anaerobic respiration. Maintaining a hypoxic microenvironment within the joint cavity protects against OA by stabilizing hypoxia-inducible factor 1α.^12^ Similarly, using a *Col11a1*-overexpressed synovial mesenchymal stem cell organoid to upregulate glycolysis in chondrocytes can effectively suppress cellular senescence and prevent cartilage degeneration.^13^ However, when OA progresses in vivo, metabolic reprogramming occurs, shifting from glycolysis toward the tricarboxylic acid (TCA) cycle to generate more adenosine triphosphate (ATP) for cell proliferation and differentiation required for cartilage repair.^14^ A biphasic dedifferentiation model was constructed using single-cell multi-omics sequencing technologies, revealing that early-stage impaired chondrocytes exhibit a glycolytic phenotype before transitioning to aerobic respiration.^15^ Targeting the SIRT3-COX4I2 axis reinvigorates oxidative phosphorylation (OXPHOS)-mediated energy synthesis to prevent cartilage degeneration.^16^ Three regulated rate-limiting enzymes exist in glycolysis: hexokinase (HK), pyruvate kinase (PK), and phosphofructokinase. Pyruvate kinase M2 (PKM2) is an important enzyme in the final stage of glycolysis and a catalyst for ATP synthesis.^17^ PKM2 degradation, mediated by sequestosome 1, inhibits the production of inflammatory mediators in macrophages, thereby mitigating synovial inflammation.^18^ Nevertheless, the specific role of PKM2 in sustaining energy synthesis in chondrocytes and the underlying mechanism remain elusive.

In this study, we assessed the role of dynamic mitophagy in the progression of chondrocyte impairment and cartilage degeneration. Subsequently, the role of PINK1 in regulating cartilage degeneration was investigated in vivo and in vitro using PINK1-overexpressing and knockout models. Mechanistically, following SIRT3-mediated deacetylation modification, activated PINK1 not only induces ubiquitin-dependent mitophagy to eliminate damaged mitochondria for mitochondrial self-renewal but also directly phosphorylates PKM2 and prevents its nuclear translocation, thereby increasing energy production by shifting from glycolysis to aerobic respiration. Finally, the protective role of the SIRT3-PINK1-PKM2 axis against OA was demonstrated in vivo using SIRT3-PINK1 double-knockout mice.

## Results

### Mitochondrial damage and maladaptive mitophagy were implicated in OA progression

To investigate the role of mitochondrial dynamics and mitophagy in the pathological process of OA, we initially collected articular cartilage samples from patients undergoing total knee arthroplasty (TKA). Compared to the lateral tibial plateau’s healthy cartilage, the medial tibial plateau’s damaged cartilage exhibited a substantial loss of C-ECM (Fig. S1A–G). The accumulation of damaged mitochondria is hazardous to cells, necessitating the engagement of mitophagic components such as the classical PINK1-PRKN for efficient cellular waste removal. Immunohistochemistry revealed a significant reduction in the expression levels of PINK1 and PRKN in damaged cartilage, suggesting potential mitophagy impairment (Fig. S1H–I). Mitochondria architecture staining showed a decreased branch length and footprints (Fig. S2A). JC-1 staining revealed a decrease in the ratio of aggregates to monomers upon in damaged cartilage, indicating impaired mitochondrial membrane potential (MMP) (Fig. S2B). Immunofluorescence assays demonstrated that insufficient PINK1 recruitment to the mitochondria resulted in a blocked mitophagy (Fig. S2C). Colocalization of LC3B with lysosomes or with P62 confirmed a reduced autophagic flux during the progression of OA (Fig. S2D, E). To visualize mitophagy, we used a mitophagy detection kit on human chondrocytes. The reduced red fluorescence intensity and the diminished co-occurrence of red or green signals suggested impeded formation of mitophagosomes and mitolysosomes (Fig. S2F). Downstream mitophagy-associated factors, including PRKN and LC3B II/I, were downregulated, whereas P62 was upregulated (Fig. S2G–I). These findings suggested the presence of aberrant mitochondrial morphology and impaired mitochondrial function in chondrocytes following OA.

**Fig. 1:**
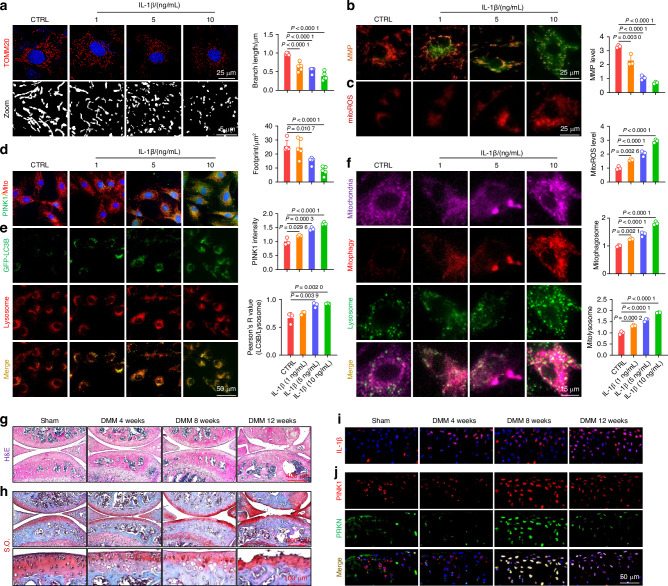
Mitochondrial damage and maladaptive mitophagy were implicated in OA progression. **a** Chondrocytes were treated with interleukin-1β (IL-1β) at varying concentrations and stained for translocase of outer mitochondrial membrane 20 (TOMM20) to measure mitochondrial branch length and footprint. Images were captured using a 100x, 1.5 NA oil immersion objective on the highly intelligent and sensitive SIM (HIS-SIM) system and subsequently processed with Wiener deconvolution. Quantification of the branch length and footprint of mitochondria was performed with mitochondrial network analysis (MiNA) toolset (*n* = 5). **b** Mitochondrial membrane potential (MMP) assessed using JC-1 staining and fluorescence microscopy in IL-1β-treated chondrocytes (*n* = 3). **c** Analysis of the representative fluorescence of mitoROS in IL-1β-treated chondrocytes (*n* = 3). **d** Co-staining of mitochondria and PINK1 using immunofluorescence (*n* = 3). **e** Evaluation of LC3B and lysosome colocalization in IL-1β-treated chondrocytes (*n* = 3). **f** Analyses of mitochondria, mitophagosomes, and lysosome colocalization in IL-1β-treated chondrocytes (*n* = 3). **g**, **h** Hematoxylin and eosin (H&E) and Safranin O (S.O.) staining of Sham and destabilization of the medial meniscus (DMM) mice at 4, 8, and 12 weeks post-surgery. **i** In vivo representative images of IL-1β-positive chondrocytes via immunofluorescence in Sham and DMM mice. **j** In vivo immunofluorescence representative images of PINK1- and PRKN-positive chondrocytes in Sham and DMM mice. The values represent mean ± standard deviation (SD). Statistically significant differences are indicated by *P* < 0.05 between the indicated groups

Murine-derived articular chondrocytes were subsequently pretreated with a commonly used cytokine interleukin-1β (IL-1β) to induce C-ECM degradation in vitro. The reverse transcription polymerase chain reaction and western blot assays confirmed the presence of disrupted C-ECM in IL-1β-treated chondrocytes (Fig. S3A–C). Since mitochondria serve as the cellular “powerhouses” for supplying energy through oxidative phosphorylation (OXPHOS), the activities of respiratory chain enzymes were assessed. In the presence of 10 ng/mL of IL-1β, the activities of the five major respiratory chain complexes were inhibited (Fig. S3D). In the chondrocytes of IL-1β-treated, both the mitochondrial branch length and footprint were significantly reduced (Fig. 1a). Moreover, IL-1β significantly reduced the levels of translocase of inner mitochondrial membrane 23 (TIM23) and translocase of outer mitochondrial membrane 20 (TOMM20) (Fig. S3E). Imbalanced mitochondrial dynamics induced by IL-1β treatment, characterized by reduced mitochondrial fusion and increased fission, confirming that the mitochondrial morphological structures were compromised (Fig. S3F). Mitochondrial functions were impaired by IL-1β, as indicated by decreased MMP and increased accumulation of mitochondrial reactive oxygen species (ROS) (Fig. 1b, c). Interestingly, unlike the completely impaired human chondrocytes, the acute inflammatory attack induced by IL-1β leaded to a compensatory increase in PINK1 within mitochondria (Fig. 1d), thereby activating a downstream mitophagic signaling cascade (Figs. 1e, f and S3G-J).

Subsequently, to investigate the role of PINK1-induced mitophagy in vivo, an OA animal model was developed via the surgical destabilization of the medial meniscus (DMM). Cartilage degeneration and subchondral bone sclerosis were observed during OA progression (Figs. 1g, h and S4A–C). Additionally, C-ECM metabolism was decoupled, as indicated by the downregulation of type II collagen (COLII) and the upregulation of matrix metallopeptidase 13 (MMP13) (Fig. S4D, E). The expression level of IL-1β within the articular cartilage gradually increased as the degenerative process progressed, confirming its strong correlation with OA (Figs. 1i and S4F). In contrast to the positive correlation observed between mitophagy activity and IL-1β concentration in vitro, the expression of PINK1 and PRKN-positive chondrocytes in vivo increased during disease progression but decreased at the end-stage, indicating a compensatory mechanism breakdown (Figs. 1j and S4G). Overall, cartilage degeneration is associated with mitochondrial impairment in chondrocytes, whereas mitophagy activation serves as a compensatory protective mechanism for eliminating damaged mitochondria.

### Targeting PINK1 activates mitophagy to eliminate defective mitochondria and facilitate mitochondrial self-renewal

**Fig. 2:**
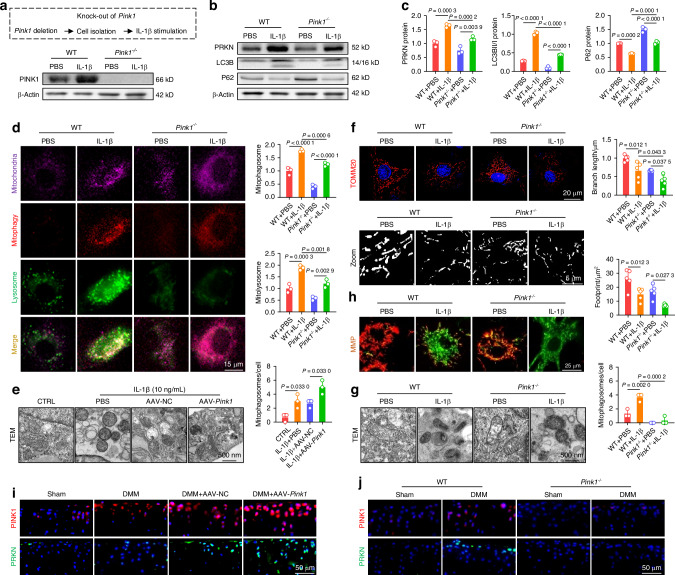
Targeting PINK1 activated mitophagy to eliminate defective mitochondria and facilitate mitochondrial self-renewal. **a** Protein levels confirm *Pink1* knockout in mice. **b**, **c** *Pink1* depletion downregulates proteins involved in the mitophagy pathway (*n* = 3). **d** Mitophagy fluorescence indicates reduced mitophagy activity following *Pink1* deficiency (*n* = 3). **e** Transmission electron microscopy (TEM) images of mouse chondrocytes following Pink1 overexpression (*n* = 3). **f** Mitochondrial branch length and footprint measurement via TOMM20 immunofluorescence post-*Pink1* deficiency (*n* = 5). **g** TEM images of mouse chondrocytes following *Pink1* deletion (*n* = 3). **h** MMP changes following *Pink1* deficiency (*n* = 3). **i** Increased cells positive for PINK1-PRKN axis-related proteins following AAV-mediated *Pink1* overexpression in the joint cavity. **j** Decreased cells positive for PINK1-PRKN axis-related proteins following *Pink1* knockout. The values represent mean ± SD. Statistically significant differences are indicated by *P* < 0.05 between the indicated groups

To investigate the impact of PINK1 on morphology and function, murine chondrocytes were pretreated with 10 ng/mL of IL-1β and subsequently transfected with the adeno-associated virus (AAV-*Pink1*). PINK1 overexpression was efficient at the transcriptional and translational levels (Fig. S5A, S5D), resulting in a significant increase in recruited PINK1 toward mitochondria (Fig. S6A). Importantly, PINK1 overexpression activated the PRKN-P62-LC3B pathway cascade, thereby facilitating autophagic flux (Figs. S5B-C and S6B). To further elucidate the impact of PINK1 deficiency on chondrocyte mitophagy, we generated *Pink1-*knockout (*Pink1*^*–/–*^) mice using clustered regularly interspaced short palindromic repeats and CRISPR-associated protein 9 (CRISPR-Cas9) technology (Fig. S5E). The successful in vivo deletion of the *Pink1* gene was confirmed using an immunoblotting assay and fluorescence colocalization (Figs. 2a and S6C). Furthermore, the chondrocytes of *Pink1*^*–/–*^ mice were collected and treated with 10 ng/mL of IL-1β. The deletion of *PINK1* resulted in a downregulation of proteins associated with mitophagy (Figs. 2b-c and S6D). The colocalization staining of LC3B and lysosomes or P62 revealed that *PINK1* overexpression facilitated the fusion between autophagosomes and lysosomes, whereas *PINK1* deletion impeded this process (Fig. S6E-H). Specifically, the mitophagy detection assay demonstrated that AAV-*Pink1*-transfected chondrocytes exhibited activated mitophagic flow, whereas chondrocytes isolated from *Pink1*^*–/–*^ mice displayed suppressed mitophagy (Figs. 2d and S7A). These findings underscore the critical role of PINK1 in regulating dynamic mitophagy. The expression levels of mitochondrial membrane proteins and mitochondrial ultrastructure observed by transmission electron microscope (TEM) revealed that PINK1 overexpression-induced mitophagy enhanced the elimination of excess mitochondria. However, the removal of dead mitochondria and debris was suppressed in *Pink1*-deficient chondrocytes (Figs. 2e–g and S7B-D). Consequently, only the functional mitochondria were selectively preserved in the presence of PINK1, whereas the defective ones were eliminated. Conversely, *Pink1*^*–/–*^ chondrocytes exhibited an aggravated mitochondrial deficit due to impaired autophagic clearance (Figs. 2h and S7E, F). Furthermore, AAV-*Pink1* injection upregulated the in vivo PINK1-PRKN axis, whereas it exhibited near-complete ablation upon CRISPR-Cas9-induced *Pink1* deletion (Figs. 2i, j and S7G). Overall, targeting PINK1 facilitates the removal of damaged organelles and the restoration of mitochondrial function by activating the PINK1-PRKN-associated mitophagy signaling pathway.

### Genetic overexpression or global knockout of PINK1 impacts cartilage homeostasis and OA development

**Fig. 3:**
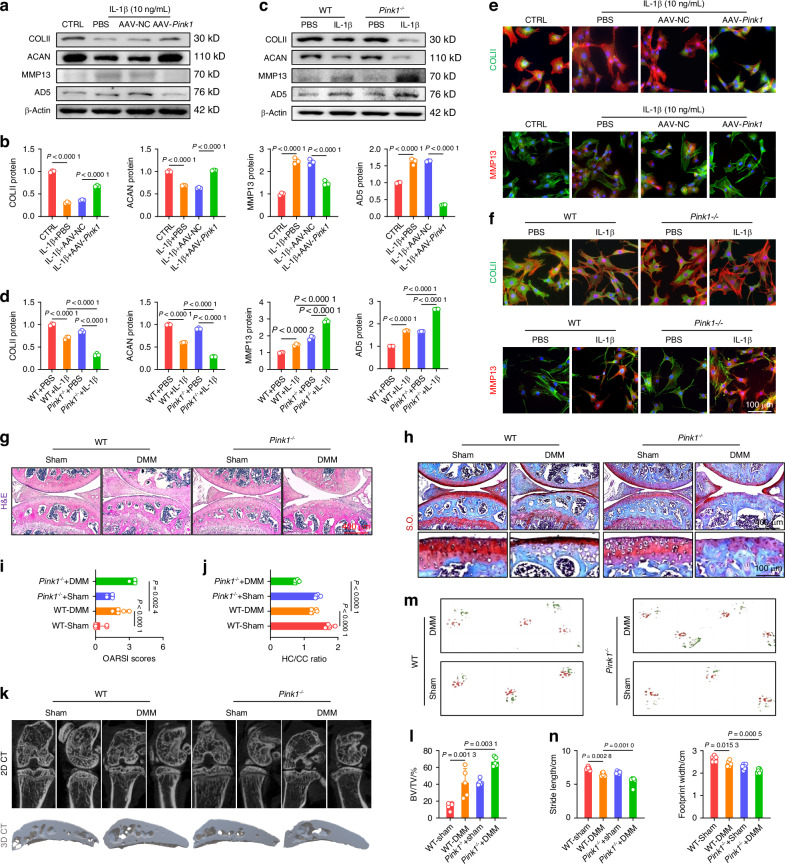
Genetic overexpression or global knockout of *Pink1* impacted cartilage homeostasis and OA development. **a**, **b** Western blot analysis of COLII, ACAN, MMP13, and ADAMTS5 protein levels following *Pink1* overexpression (*n* = 3). **c**, **d** Western blot analysis of COLII, ACAN, MMP13, and ADAMTS5 protein levels following *Pink1* deficiency (*n* = 3). **e**, **f** Cellular immunofluorescence for COLII or MMP13 in the context of *Pink1* overexpression and deficiency, respectively. **g**, **h** Representative images of H&E and S.O. staining following *Pink1* knockout. **i**, **j** Quantitative analysis of osteoarthritis research society international (OARSI) scores and the ratio of hyaline cartilage (HC) to calcified cartilage (CC) (*n* = 5). **k** Micro-computed tomography (μ-CT) imaging analyses comparing wild-type and *Pink1*-knockout (*Pink1*^*–/–*^) mice before and after surgery. **l** Quantitative analysis of trabecular bone volume fraction (BV/TV) of wild-type and *Pink1*^*–/–*^ mice (*n* = 5). **m**, **n** Gait analysis and quantification of stride length and footprint width of wild-type and *Pink1*^*–/–*^ mice (*n* = 6). The values represent mean ± SD. Statistically significant differences are indicated by *P* < 0.05 between the indicated groups

To investigate the potential protective effects of PINK1 on cartilage degeneration, murine chondrocytes were assessed for C-ECM composition in an IL-1β-induced OA microenvironment in vitro. At the mRNA and protein levels, PINK1 overexpression not only increased COLII and aggrecan (ACAN) secretion but also suppressed the activities of proteinases, including MMP13 and ADAM metallopeptidase with thrombospondin type 1 motif 5 (ADAMTS5) (Figs. 3a, b and S8A). Conversely, *Pink1*-deficient chondrocytes exhibited disorganized C-ECM metabolism, characterized by reduced ECM marker synthesis and hyperactivation of matrix-degrading enzymes (Figs. 3c, d and S8B). Immunofluorescence staining revealed that AAV-*Pink1* treatment restored the balanced expression of COLII and MMP13 within chondrocytes, which deteriorated following *Pink1* knockout (Figs. 3e, f and S8C, D). Subsequently, a comprehensive evaluation was conducted to assess the effects of PINK1 overexpression or knockout on OA phenotype in vivo. Articular cartilage degeneration and hyaline cartilage (HC) layer thickness were significantly reduced following surgical DMM. Intra-articular administration of AAV-*Pink1* decreased cartilage degeneration, as indicated by a reduction in OARSI scores and an increase in the HC to calcified cartilage (CC) ratio (Fig. S9A–D). Conversely, despite the absence of alterations in cartilage phenotypes between wild-type (WT) and *Pink1*^*–/–*^ mice, PINK1 deletion considerably worsened cartilage damage compared with WT mice following surgical DMM (Fig. 3g–j). OA progression was characterized not only by superficial disruption of cartilage integrity but also by subchondral bone sclerosis and osteophyte formation. The micro-computed tomography imaging revealed that the DMM-induced abnormal increase in local bone mass at the subchondral bone plate (cortical bone-like region) was improved in AAV-*Pink1* mice, whereas it was further aggravated in *Pink1*^*–/–*^ mice (Figs. 3k, l and S9E–H). Subsequently, the joint mobility function was assessed using a footprint test, revealing substantial impairment in DMM mice as indicated by significant reductions in stride length and footprint width parameters. PINK1 modification confers protection against the reduced mobility caused by joint capsule fibrosis and pain (Figs. 3m, n and S9I, J). Overall, PINK1 deficiency promotes OA progression by altering C-ECM homeostasis and impairing physiological structures and exercise capacity.

### SIRT3-PINK1 axis activates mitophagy and cartilage anabolism via deacetylation to counteract OA

**Fig. 4:**
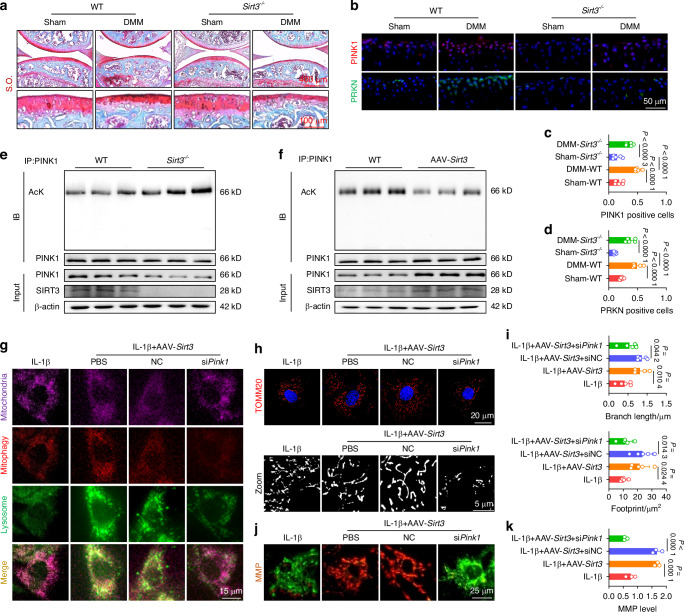
SIRT3-PINK1 axis activated mitophagy and cartilage anabolism via deacetylation to counteract OA. **a** S.O. staining in *Sirt3*-knockout (*Sirt3*^*–/–*^) mice. **b**–**d** Representative images and quantification of PINK1- or PRKN-positive chondrocytes in vivo for wild-type and *Sirt3*^*–/–*^ mice (*n* = 5). **e** Detection of total protein content and acetylation level of PINK1 in wild-type and *Sirt3*-deficient chondrocytes using co-immunoprecipitation (Co-IP) assay. **f** Detection of total protein content and acetylation level of PINK1 in wild-type and *Sirt3*-overexpressing chondrocytes using Co-IP assay. **g** SIRT3-induced mitophagy is reduced by si*Pink1* transfection. **h**, **i** Impaired SIRT3-mediated mitochondrial length and footprint improvements following si*Pink1* transfection (*n* = 5). **j**, **k** Impaired SIRT3-mediated MMP improvements following si*Pink1* transfection (*n* = 3). The values represent mean ± SD. Statistically significant differences are indicated by *P* < 0.05 between the indicated groups

Because PINK1 plays a critical role in regulating the clearance of damaged mitochondria and maintaining a balanced C-ECM metabolism, we further investigated the upstream signaling molecule responsible for PINK1 activation. Given the crucial role of mitochondrial deacetylases in maintaining mitochondrial homeostasis homeostasis, we examined the expression levels of three mitochondrial deacetylases: SIRT3,^19^ SIRT4,^20^ and SIRT5.^21^ Among these, SIRT3 showed the most significant changes in response to IL-1β stimulation (Fig. S10A). Meanwhile, human damaged cartilage exhibited increased acetylation of PINK1, suggesting a potential deacetylation-mediated relationship between SIRT3 and PINK1 (Fig. S10B). Subsequently, we treated IL-1β-impaired chondrocytes with a specific SIRT3 enzymatic inhibitor (3-TYP) in the presence or absence of AAV-*Pink1*. Inhibition of SIRT3 activity partially attenuated the chondroprotective effects and mitophagy activation mediated by PINK1 (Fig. S10C–E). Interestingly, overexpression or deletion of PINK1 did not alter the expression of SIRT3 in both in vitro and in vivo experiments, suggesting a potential unidirectional regulatory effect of SIRT3 on PINK1 (Fig. S11A, B). To elucidate the underlying mechanisms, SIRT3-knockout (*Sirt3*^*–/–*^) mice were generated utilizing CRISPR/Cas9 technology (Fig. S11C). In vivo SIRT3 deletion not only facilitated cartilage degeneration and C-ECM loss but also aggravated structural remodeling in the subchondral bone (Figs. 4a and S12A–C). The impaired motor functions were further aggravated in DMM mice by SIRT3 deficiency, as indicated by a reduction in stride length and footprint width (Fig. S12D). Importantly, surgical DMM significantly upregulated the expression of the PINK1-PRKN axis in articular cartilage; however, SIRT3 deletion significantly inhibited the induction of activated mitophagy (Fig. 4b–d). The co-immunoprecipitation (Co-IP) assay confirmed a decrease in the overall protein content of PINK1 and an increase in PINK1 acetylation in *Sirt3*-deficient chondrocytes (Fig. 4e). Conversely, SIRT3 overexpression effectively maintained PINK1 levels and enzymatic activities in a deacetylase-independent manner (Fig. 4f). Subsequently, IL-1β-stimulated chondrocytes were treated with AAV-*Sirt3* and si*Pink1* to elucidate the SIRT3-PINK1 interaction. PINK1 knockdown inhibited SIRT3-induced mitophagy, as evidenced by reduced colocalization of LC3B with lysosomes and P62, as well as diminished mitophagy staining (Figs. 4g and S13A, B). Treatment with si*Pink1* significantly reduced PINK1 recruitment to damaged mitochondria (Fig. S13C) and suppressed the downstream PRKN-P62-LC3B cascade (Fig. S13D). SIRT3-mediated improvements in mitochondrial morphology and function were compromised following si*Pink1* transfection, as evidenced by mitochondrial structural disruption (Figs. 4h, i and S13E) and functional impairment (Fig. 4j, k). Regarding C-ECM metabolism, the SIRT3-PINK1 axis disruption significantly decreased cartilage anabolism while enhanced catabolic activities, aggravating the OA phenotype (Fig. S14A, B).

**Fig. 5:**
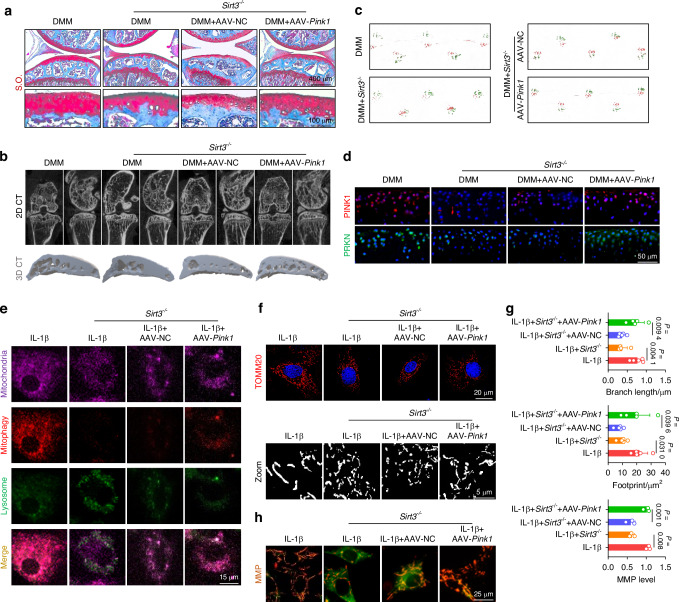
Overexpression of *Pink1* ameliorated the decrement in mitophagy engendered by *Sirt3* knockout to impede the progression of OA. **a** S.O. staining in *Sirt3*-knockout (*Sirt3*^*–/–*^) mice following AAV-mediated *Pink1* overexpression. **b** μ-CT imaging analysis comparing *Sirt3*^*–/–*^ mice before and after overexpression of *Pink1*. **c** Gait analysis of *Sirt3*^*–/–*^ mice before and after overexpression of *Pink1*. **d** In vivo immunofluorescence representative images of PINK1- or PRKN-positive chondrocytes for *Sirt3*^*–/–*^ mice before and after overexpression of *Pink1*. **e** SIRT3-induced mitophagy is rescued by overexpression of *Pink1*. **f**–**g** Mitochondrial length and footprint measurement via TOMM20 immunofluorescence in *Sirt3* knockout cells following the overexpression of *Pink1* (*n* = 5). **h** MMP changes in *Sirt3* knockout cells following the overexpression of *Pink1* (*n* = 3). The values represent mean ± SD. Statistically significant differences are indicated by *P* < 0.05 between the indicated groups

To clarity the role of the SIRT3-PINK1 axis in activating mitophagy and protecting C-ECM, we further overexpressed PINK1 in SIRT3-deficient chondrocytes. Intra-articular injection of AAV-*Pink1* mitigated cartilage degradation and inhibited subchondral osteophyte formation (Figs. 5a, b and S15A–C). Impaired lower-limb motor function in *Sirt3*-deficient mice was significantly improved following treatment with AAV-*Pink1* (Figs. 5c and S15D). Importantly, supplementation of PINK1 reactivated the PINK1-PRKN axis and restored mitochondrial autophagy in the absence of SIRT3, resulting in the promotion of mitochondrial self-renewal and the maintenance of mitochondrial health (Figs. 5d–h and S16A–E). Consequently, the suppression of C-ECM synthetic capacity caused by SIRT3 deletion was recovered through the overexpression of PINK1 (Fig. S17A, B). Together, SIRT3 effectively activates PINK1 by targeted deacetylation to safeguard against mitochondrial damage and accumulation, hence counteracting cartilage degeneration.

### PINK1 directly phosphorylates PKM2 and inhibits its nuclear translocation to promote energy production

**Fig. 6:**
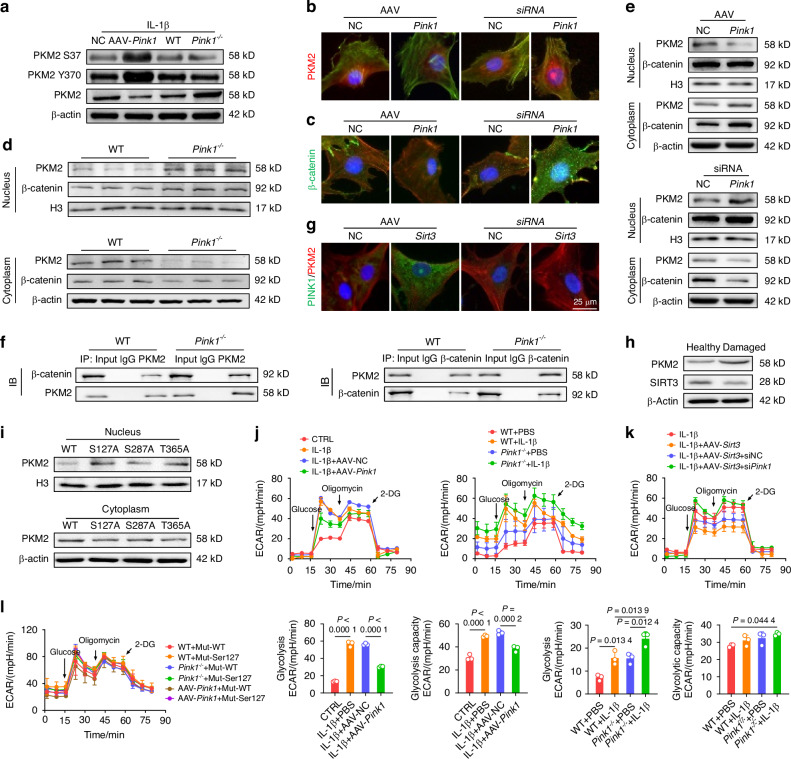
PINK1 directly phosphorylated PKM2 and inhibited its nuclear translocation to promote energy production. **a** Impact of *Pink1* deletion and overexpression on PKM2 protein levels and phosphorylation at Tyr370 and Ser37. **b**, **c** Immunofluorescence assessment of PKM2 and β-catenin nuclear translocation following PINK1 modulation. **d** Quantitative analysis of PKM2 protein distribution in wild-type and *Pink1*^*–/–*^ mice using nucleocytoplasmic fractionation. **e** Assessment of the nuclear-cytoplasmic distribution of PKM2 and β-catenin upon PINK1 overexpression and si*Pink1* treatment. **f** Interaction between PKM2 and β-catenin after *Pink1* knockout using Co-IP assay. **g** Observation of colocalization of PKM2 and PINK1 following overexpression or knockdown of *Sirt3*. **h** Protein levels of PKM2 and SIRT3 in healthy or damaged cartilage tissues from OA patients. **i** Analysis of the impacts of PKM2 mutations at three phosphorylation sites (Ser127, Ser287, and Thr365) on its subcellular distribution through nucleocytoplasmic fractionation. **j** Extracellular acidification rate (ECAR) analysis following *Pink1* overexpression or knockout (*n* = 3). **k** ECAR analysis following *Sirt3* overexpression and subsequent si*Pink1* transfection (*n* = 3). **l** Effect of PKM2 mutation at the Ser127 phosphorylation site on ECAR Levels in wild-type, PINK1-overexpressing, and PINK1-deficient chondrocytes (*n* = 3). The values represent mean ± SD. Statistically significant differences are indicated by *P* < 0.05 between the indicated groups

To further elucidate the underlying mechanism by which PINK1 inhibits C-ECM disintegration and identifies downstream effector molecules, chondrocytes derived from mice were subjected to phosphoproteomic sequencing. PINK1 deletion resulted in the identification of 61 differentially expressed phosphorylated peptides (|logFC | > 1 and *P* < 0.05), corresponding to 53 proteins (Fig. S18A). According to gene ontology (GO) enrichment analysis, PINK1-mediated phosphorylation modification significantly disrupted glycolysis-related biological processes, including canonical glycolysis, glucose catabolic process to pyruvate, and glycolytic process through glucose-6-phosphate (Fig. S18B–E). Similarly, the Kyoto encyclopedia of genes and genomes (KEGG) analysis provided additional evidence supporting the significant enrichment of glycolysis/gluconeogenesis and metabolic pathways (Fig. S18F-G). Based on the previous integration of phosphoproteomic results (logFC = −1.51 and *P* = 0.019) and the critical role of PKM2 in regulating metabolic homeostasis,^22^ we postulated that PKM2 serves as a direct downstream effector molecule in response to PINK1-mediated phosphorylation modification. Co-IP experiments corroborated this hypothesis by demonstrating the binding of endogenous PINK1 and PKM2 (Fig. S19A). Furthermore, PINK1 deletion upregulated the total protein levels of PKM2 while downregulating the phosphorylated protein levels at the identified modifying phosphorylation sites (Ser37 and Tyr370) (Figs. 6a and S19B). The PKM2 tetramer is an active pyruvate kinase that regulates cellular respiration, whereas the PKM2 dimer functions as a protein kinase by translocating to the nucleus and initiating gene transcription.^23^ Immunofluorescence staining revealed that PINK1 knockdown facilitated PKM2 translocation to the nucleus (Fig. 6b). Given the recent identification of β-catenin as a crucial downstream effector of PKM2,^24^ we investigated the interaction between PKM2 and β-catenin in response to PINK1. The immunoblotting assays and immunofluorescence staining revealed that PINK1 deletion stabilized β-catenin in the nucleus (Fig. 6c) and facilitated the formation of a complex between β-catenin and PKM2, while overexpression of PINK1 inhibited the translocation of PKM2 to the nucleus and its interaction with β-catenin (Figs. 6d–f and S19C). Additionally, the interaction of PINK1 with PKM2 was partially attenuated in the absence of SIRT3, further supporting the role of SIRT3 in regulating the PINK1-PKM2-β-catenin signaling axis (Figs. 6g and S19D). Human cartilage samples also demonstrated a decreased expression of SIRT3 alongside an increased expression of PKM2 in damaged cartilage (Fig. 6h).

Notably, the phosphorylation at Ser37^25^ and Tyr370^26^ induce dimerization of PKM2 and its subsequent translocation into the nucleus, which contradicts our findings. Consequently, we hypothesized that other PINK1-mediated phosphorylation sites may facilitate the formation of PKM2 tetramers. After integrating phosphorylation sequencing data, three potential sites (Ser127, Ser287, and Thr365) were selected for mutant plasmid construction. Ser127 mutation significantly reduced the PINK1-induced translocation of PKM2 to the nucleus, demonstrating that PINK1 promotes the formation of PKM2 tetramers by directly phosphorylating PKM2 at the Ser127 site (Fig. 6i). Finally, the glycolytic activity and cellular energy synthesis levels were evaluated using the Seahorse assay. Genetic knockdown of PKM2 results in decreased energy generation, attributable to its critical role in the glycolysis pathway (Fig. S20A). PINK1 overexpression promotes a metabolic shift from anaerobic glycolysis to aerobic respiration, thereby decreasing glycolytic activity and capacity while enhancing ATP and NADH production (Fig. S20B). However, the deletion of PINK1 further augmented glycolytic activity. (Fig. 6j). Meanwhile, SIRT3 activation inhibited anaerobic respiration, increasing metabolic capacity and bioenergetic efficiency; however, PINK1 knockdown reversed these beneficial effects (Figs. 6k and S20C). Specifically, the mutations at the Ser127 phosphorylation site of PKM2 protected against PINK1-mediated inhibition of nuclear import, thereby promoting glycolytic activity (Figs. 6l and S20D). Overall, PINK1 interacted with and phosphorylated PKM2 at the Ser127 site, resulting in PKM2 redistribution and metabolic reprogramming.

### Double-knockout of SIRT3 and PINK1 in vivo aggravates cartilage-bone degeneration and motor function impairment

**Fig. 7:**
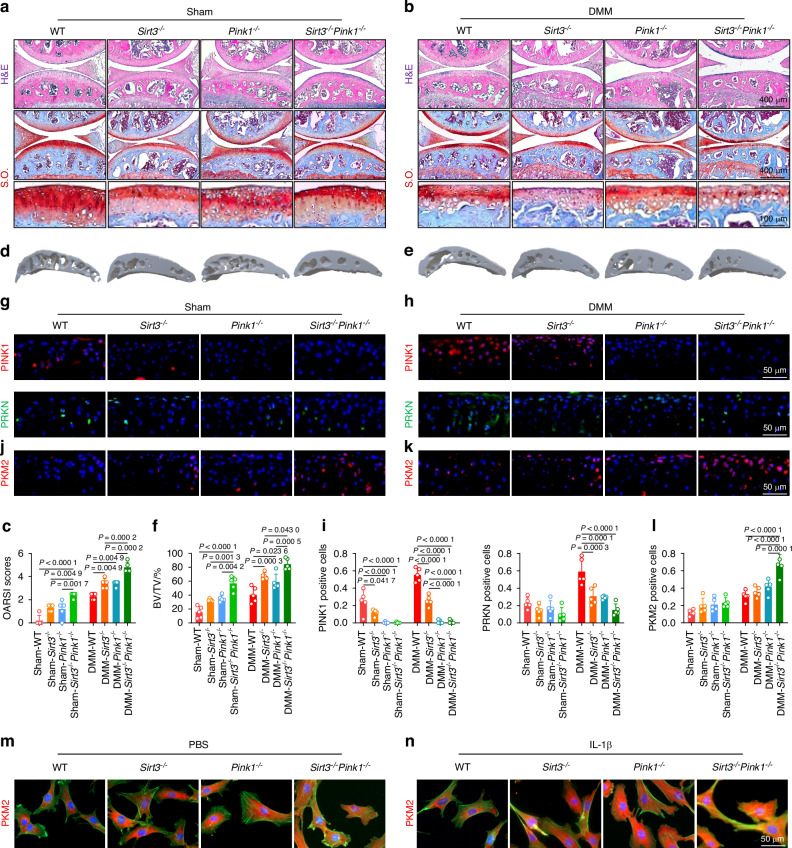
Double-knockout of *Sirt3* and *Pink1* in vivo aggravated cartilage-bone degeneration and motor function impairment. **a**–**c** H&E and S.O. staining in *Sirt3*^*–/–*^ *Pink1*^*–/–*^ mice compared with wild-type, *Sirt3*^*–/–*^, and *Pink1*^*–/–*^ mice before and after surgery (*n* = 5). **d**–**f** μ-CT imaging analyses in *Sirt3*^*–/–*^ *Pink1*^*–/–*^ mice compared with wild-type, *Sirt3*^*–/–*^, and *Pink1*^*–/–*^ mice before and after surgery (*n* = 5). **g**–**l** In vivo Immunofluorescence representative images of PINK1-, PRKN-, or PKM2-positive chondrocytes in *Sirt3*^*–/–*^ *Pink1*^*–/–*^ double-knockout mice before and after surgery compared with other genotypes within the same experimental group (n = 5). **m**, **n** Representative immunofluorescence microscopy images illustrating PKM2 nuclear translocation in chondrocytes derived from *Sirt3*^*–/–*^ *Pink1*^*–/–*^ mice compared with wild-type, *Sirt3*^*–/–*^, and *Pink1*^*–/–*^ mice before and after IL-1β treatment. The values represent mean ± SD. Statistically significant differences are indicated by *P* < 0.05 between the indicated groups

**Fig. 8:**
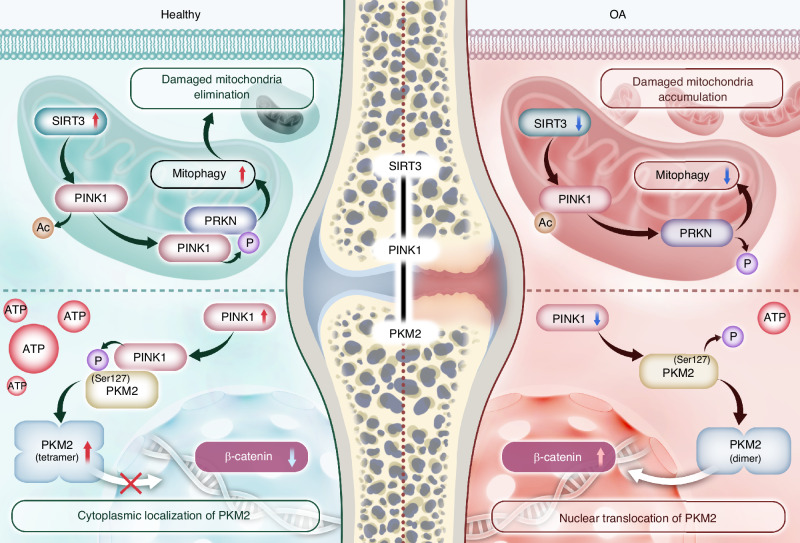
An overview of the regulatory mechanisms of SIRT3-PINK1-PKM2 in OA pathogenesis. SIRT3 directly deacetylates PINK1, enhancing its activities. Subsequently, PRKN is recruited and initiates mitophagy flux to eliminate damaged mitochondria. Additionally, PINK1 directly phosphorylates PKM2 at the Ser127 site to preserve its tetrameric conformation and prevent nuclear translocation, thereby facilitating the metabolic shift toward efficient energy production

Considering the aforementioned findings regarding the chondroprotective effects of the SIRT3-PINK1 axis, we investigated whether the simultaneous deficiency of SIRT3 and PINK1 could result in increased cartilage degeneration and an OA phenotype. We generated *Sirt3*^*–/–*^ *Pink1*^*–/–*^ double-knockout mice (Fig. S21A). The *Sirt3*^*–/–*^ *Pink1*^*–/–*^ mice exhibited significant articular cartilage wear and an upshift of the tidemark line compared with the *Sirt3*^*–/–*^ and *Pink1*^*–/–*^ mice. The simultaneous deletion of SIRT3 and PINK1 in OA mice after surgical DMM considerably aggravated cartilage degeneration (Fig. 7a–c). Consequently, the delicate balance between C-ECM anabolism and catabolism was completely disrupted following the deletion of both genes (Fig. S22A–C). SIRT3 or PINK1 deficiency resulted in mild subchondral bone sclerosis, whereas the double-knockout mice exhibited significant osteophyte formation compared with the WT mice. As OA progressed, *Sirt3*^*–/–*^ *Pink1*^*–/–*^ double-knockout mice exhibited an extreme pathological condition with a complete mineralized structure (Figs. 7d–f and S22D–F). Importantly, the double-knockout mice exhibited a significantly reduced locomotor function, indicating the dominant protective effects of the SIRT3-PINK1 axis on overall joint homeostasis (Fig. S22G–I). Finally, the intrinsic pathway changes were examined to elucidate the mechanisms underlying the impact of SIRT3 and PINK1 on chondrocytes. In response to the pathological environment of OA, the PINK1-PRKN axis transitioned from a dormant to an active state to eliminate damaged organelles. However, SIRT3 and PINK1 deletion rendered this self-protective mechanism ineffective (Fig. 7g–i). Further investigation into glycolysis signaling revealed that the double deletion not only increased the concentration of total PKM2 in cartilage (Fig. 7j–l) but also facilitated its nuclear translocation (Fig. 7m, n), indicating a conformational transition from a tetramer to dimers in its nonphosphorylated state. Overall, the simultaneous knockout of SIRT3 and PINK1 in vivo further disrupted cartilage function and aggravated joint damage Fig. 8.

## Discussion

As a cellular scavenger, autophagy is an important catabolic process in cellular housekeeping and homeostasis maintenance. Pharmacological inhibition of the mammalian target of rapamycin (mTOR), a critical regulator that suppresses autophagy, improves OA phenotype.^27^ Using CRISPR-Cas9 gene editing technology, the inducible deletion of mTOR specifically in cartilage resulted in enhanced autophagy signaling and significant preservation of cartilage integrity.^28^ Moreover, conditional knockout of ATG5, an essential regulator of autophagy, was associated with compromised cell survival.^29^ The Forkhead Box O (FoxO) signaling pathway functions as an upstream regulator of autophagy for OA treatment.^30^ Specifically, FoxO1^31^ and FoxO3^32^ transcription factors have been harnessed to develop potential pre-clinical drugs, such as Panobinostat.^33^ Additionally, the activation of autophagic process in chondrocytes has identified G protein-coupled receptor class C group 5 member B (GPRC5B),^34^ HECT Domain E3 Ubiquitin Protein Ligase 1 (HECTD1),^35^ lipid metabolism-related peroxisome proliferator-activated receptor γ (PPARγ),^36^ and methyltransferase-like 3 (METTL3)^37^ as potential therapeutic targets for preventing cartilage degradation. From a subcellular perspective, this study revealed that mitophagy activation exerts a robust chondroprotective effect in a ubiquitin kinase PINK1- and E3 ubiquitin ligase PRKN-dependent manner. Elevated PINK1 expression was observed in both the monosodium iodoacetate (MIA)-induced OA model and human osteoarthritic cartilage. Deficiency in PINK1 expression was associated with reduced cartilage damage and attenuated pain behaviors.^8^ Intriguingly, in the DMM-induced in vivo OA model, we observed an increased expression of PINK1 in cartilage at the early stage, which was followed by a decreased expression at the late stage. Together with the controversial findings regarding PINK1 levels in proinflammatory cytokine-stimulated mouse chondrocytes and naturally degenerated human cartilage, we proposed that the dynamic expression of PINK1 is regulated by an early compensatory mechanism followed by a late decompensatory process. Therefore, a mitophagy-targeted therapeutic strategy should be tailored to the distinct phases of disease progression to achieve precision medicine. In addition to PINK1-ubiquitin-mediated mitophagy, the mitochondrial autophagy receptor BNIP3-induced recognition of autophagosomes has been implicated in C-ECM pathogenesis. BNIP3 regulation in the chondroid nucleus pulposus (NPs) modulates mitochondrial function and metabolic status^38^ by adapting to the local hypoxic microenvironment.^39^ NLR family member X1 (NLRX1)-mediated hyperactivation of mitophagy accelerates NP senescence and IVDD progression, suggesting that mitophagy exhibits a dual role in cartilage degeneration.^40^ Importantly, significant progress has been achieved in the development of specific pharmacological agents that enhance mitophagy, thereby considerably improving cartilage functionality. Supplementation of physiological metabolite with α-ketoglutarate^41^ improved the OA phenotype by modulating mitophagy. A clinical trial demonstrated the remarkable potential of commodified urolithin A (Mitopure®) in effectively delaying joint damage, providing significant support for the therapeutic application of mitophagy in OA.^42^

Physiologically, chondrocytes in hypoxic microenvironments sustain their metabolic activities via glycolysis to generate energy for cellular functions. A comprehensive multi-omics analysis revealed metabolic-related gene dysregulation in OA chondrocytes, characterized by a metabolic shift toward increased glycolysis at the expense of mitochondrial respiration.^43^ Meanwhile, the inefficient energy production of OA-induced elevated glycolysis in chondrocytes was associated with reduced cellular ATP synthesis, impaired mitochondrial function, and inhibited C-ECM synthesis.^44^ A recent study revealed that hemoglobin serves a novel non-erythrocyte role in chondrocytes. Furthermore, severe hypoxia-induced elevated glycolysis is closely associated with extensive cellular apoptosis.^45^ Deleting lactate dehydrogenase A (LDHA) in chondrocytes effectively mitigated OA by suppressing cellular metabolism toward glycolysis.^46^ In this study, SIRT3-PINK1 counteracted the upregulated glycolytic activities in chondrocytes, resulting in increased ATP generation and restored metabolic homeostasis. A lack of platelet-derived growth factor-BB (PDGF-BB) can inhibit glycolysis, resulting in pyroptosis in NPs and aggravating IVDD.^47^ This demonstrates the multifaceted role of glycolysis in musculoskeletal disorders. In addition to glycolysis in chondrocytes, inflammatory exosomes increase glycolytic activities in synovial macrophages, promoting macrophage polarization toward the M1 phenotype and contributing to OA.^48^ Clinical evidence from patients revealed that increased glycolysis in fibroblast-like synoviocytes (FLSs) is a key driving force for macrophage infiltration into OA synovium and polarization toward an M1 phenotype.^49^ Considering that OA drives angiogenesis, the angiogenic response induced by anti-miR-125a in endothelial cells (ECs) is associated with a metabolic profile that favors glycolysis, suggesting that increased glycolysis in ECs may contribute to OA.^50^ Reprogramming cellular metabolism by modulating glycolysis holds significant potential for treating joint destruction in inflammatory arthritis, such as rheumatoid arthritis. Pharmacological inhibition of glycolysis with bromopyruvic acid directly modulates the inflammatory functions mediated by FLSs to reduce cartilage degeneration.^51^ Moreover, inhibiting lysine-specific demethylase 1 in osteoclast precursors^52^ or specifically deleting hexokinase 2 (HK2) in non-hematopoietic cells^53^ reduces arthritis severity and bone and cartilage damage by modulating glycolytic activities.

PKM2 is the final rate-limiting kinase in aerobic glycolysis, ensuring a balanced metabolic equilibrium between OXPHOS and glycolysis. Previous studies presented conflicting perspectives on the impact of PKM2 on OA. One viewpoint suggests that PKM2 knockdown impairs C-ECM secretion owing to decreased glycolysis.^54^ Another perspective argues that PKM2 inhibition protects cartilage homeostasis by reducing endoplasmic reticulum stress^55^ or inhibiting cellular senescence.^56^ A clinical observational study revealed a significant association between increased PKM2 genes in whole blood and the development of more severe pain following arthroplasty, implying that PKM2 exhibits a potentially detrimental role in OA.^57^ Our findings revealed that PINK1 protects against cartilage degeneration by modulating PKM2 phosphorylation and nuclear translocation rather than altering its total protein abundance. The tetramer-dimer transition of PKM2 is critical in biological processes, as high-activity PKM2 tetramers drive the TCA cycle,^58^ whereas dimeric PKM2 initiates gene transcription and promotes the Warburg effect by translocating to the nucleus.^59^ Pharmacological activation of PKM2 improves experimental autoimmune reactions by inducing tetramerization and inhibiting nuclear translocation.^60^ Conversely, follistatin-like protein 1 directly binds to and facilitates the nuclear translocation of dimeric PKM2, thereby enhancing glycolysis and aggravating hepatic inflammation.^61^ Our findings revealed that PINK1 phosphorylation of PKM2 induces its tetramerization and enhances OXPHOS-dependent high-efficiency energy production. Phosphorylated PKM2 exhibits a propensity to form dimers via modifications such as phosphorylation at Ser37 or Tyr105.^25,62^ The oncoprotein cancerous inhibitor of protein phosphatase 2 A phosphorylates PKM2 at Ser287, inducing PKM2 tetramer formation.^63^ We discovered an additional phosphorylation site at Ser127, which is critical in facilitating PKM2 tetramer formation and inhibiting its nuclear translocation. SIRT3-PINK1 suppressed the interaction between nuclear PKM2 and β-catenin by reducing nuclear inflow. The activation of β-catenin by tyrosine kinase Fyn can accelerate cartilage degradation.^64^ Conversely, its inhibition is beneficial for C-ECM homeostasis^65^ and joint inflammation reduction.^66^

### Limitations of the study

This study has two drawbacks. First, the significance of the SIRT3-PINK1 axis in OA progression was assessed using global gene knockout mice; thus, the effects of this intervention on other tissues cannot be excluded. Subsequent research should use mouse models with cartilage-specific deletions to elucidate the specific role of the SIRT3-PINK1-PKM2 axis in chondrocyte homeostasis. Second, we discovered that PKM2 phosphorylation sites modified by PINK1 can elicit contrasting effects, resulting in the formation of dimers or tetramers. However, further investigations are needed to elucidate the underlying mechanisms and rationale behind the selective binding of PINK1 to specific sites on PKM2.

## Materials and methods

### Isolation and culture of chondrocytes

Chondrocytes were isolated from the knee joints of mice and human patients by finely dicing the cartilage tissue, followed by prolonged incubation at 37 °C with 0.2% Type II Collagenase (Thermo Fisher Scientific, Waltham, MA, USA). After overnight digestion, undigested tissue remnants were filtered out the following day. The resulting cell suspension was then re-suspended and seeded. Primary chondrocytes were cultured in F-12 culture medium (DMEM/F-12, Thermo Fisher Scientific, Waltham, MA, USA) supplemented with 10% Fetal Bovine Serum (FBS, Thermo Fisher Scientific), 100 units/mL Penicillin, and 100 units/mL Streptomycin (Thermo Fisher Scientific) to promote cell growth and prevent microbial contamination. Cells were maintained at 37 °C in a 5% CO_2_ environment, with all subsequent experimental procedures performed using passage P1 chondrocytes.

### Cellular interventions

To investigate the impacts of varying concentrations of inflammatory stimuli on chondrocytes, we utilized 1, 5, and 10 ng/mL of human recombinant IL-1β (Thermo Fisher Scientific), alongside establishing a blank control group. To simulate an in vitro arthritic microenvironment, mouse chondrocytes were incubated with 10 ng/mL human recombinant IL-1β. Small interfering RNAs (siRNAs) were sourced from Shanghai GenePharma Co., Ltd., while adeno-associated viruses (AAVs) were obtained from Shanghai OBiO Technology Co., Ltd. Transfection of Small-interfering RNA (siRNA) was facilitated using Lipofectamine^TM^ 3000 (Thermo Fisher Scientific), followed by subsequent treatments with specific agents. To elucidate the functions of PINK1 and SIRT3, cells were transfected with either 100 nmol/L siRNA targeting *Pink1* or adeno-associated virus-*Pink1* (AAV-*Pink1*) at concentrations ranging from 10^9^ to 10^11^ genome copies per mL. Similarly, the role of SIRT3 was examined using 100 nmol/L siRNA targeting *Sirt3* or adeno-associated virus-*Sirt3* (AAV-*Sirt3*) at equivalent concentrations. Modulation of glycolysis involved transfecting cells with 100 nmol/L siRNA targeting PKM2. To manipulate phosphorylation states at specific sites, cells were transfected with 100 nmol/L of PKM2 mutants: S127A, S287A, and T365A. To examine the impact of deacetylase inhibitors on mitophagy and C-ECM metabolism, chondrocytes were treated with 3-TYP (MCE, HY-108331) at a concentration of 50 μmol/L.

### Quantitative real‑time reverse transcription‑polymerase chain reaction (RT‑PCR)

Mouse chondrocytes were seeded in six-well plates and subjected to the described interventions. Total RNA extraction was carried out using TRIzol® reagent (Thermo Fisher Scientific) followed by reverse transcription to cDNA using the RevertAid First Strand cDNA Synthesis Kit (Thermo Fisher Scientific). Quantitative reverse transcription PCR (RT-PCR) was performed with the ChamQ Blue Universal SYBR qPCR Master Mix (Vazyme, Nanjing, China) using the CF X96TM Real-Time PCR System. Transcript levels of the target genes were quantified using the comparative Ct (2^−ΔΔCt^) method. Primer sequences utilized for RT-qPCR are provided in Supplementary Table 1.

### Western blotting

Following the implementation of various interventions outlined previously, cells from the culture dish were harvested and lysed on ice for 30 minutes using NCM RIPA Buffer (NCM Biological Technology Co., Ltd, China), followed by centrifugation. The resulting supernatant was collected to assess total protein content. Protein concentrations of each sample were determined with the BCA Protein Quantification Kit (Vazyme). Subsequently, proteins were separated by sodium dodecyl sulfate-polyacrylamide gel electrophoresis (SDS-PAGE) and transferred onto a nitrocellulose membrane (Beyotime, Shanghai, China). The membrane was blocked with a blocking buffer for 30 minutes at room temperature to minimize non-specific binding, followed by washing with washing buffer. Next, the membrane was incubated overnight at 4 °C with specific primary antibodies. After three washes with the washing solution, the membrane was incubated with secondary antibodies for 1 hour at room temperature. Following another thorough wash to remove unbound secondary antibodies, immunoreactive bands were visualized using ultra-sensitive Enhanced Chemiluminescent (ECL, NCM) and captured using the SH-523 imager (Shenhua Technology, Hangzhou, China). Band intensities were quantified using Image J software (National Institutes of Health, Bethesda, MD, USA) for further analysis.

### Nuclear and cytoplasmic protein extraction

For the extraction of nuclear and cytoplasmic proteins, the Nuclear and Cytoplasmic Protein Extraction Kit (P0028, Beyotime) was employed to effectively fractionate cellular components from samples. Cells were harvested by rinsing with phosphate-buffered saline (PBS, RG-CE-10, KETU) to remove the extracellular culture medium, followed by detachment using a cell scraper. After centrifugation, the supernatant was carefully aspirated to collect the cell pellet, allowing cells to settle. To extract cytoplasmic protein, 200 µL of reagent A, supplemented with 1 mmol/L Phenylmethanesulfonyl fluoride (PMSF, ST506, Beyotime), was added per 20 µL of cell pellet. The mixture was incubated on ice for 15 minutes. Subsequently, 1 µL of reagent B was added to initiate cell lysis, followed by a brief vortex at maximum speed for 5 seconds, and further incubation on ice for 1 minute. After vortexing and centrifugation at 4 °C, 12 000 *g* for 5 minutes, the resulting supernatant containing cytoplasmic proteins was transferred to a precooled tube. For nuclear protein extraction, the residual supernatant was aspirated, and 50 µL of nuclear extraction reagent containing PMSF was added to the pellet. Intense vortexing for 30 seconds suspended the cells, followed by intermittent ice incubation and vortexing for 30 minutes to ensure efficient cell disruption and release of nuclear proteins. The nuclear proteins were pelleted by centrifugation at 4 °C, 12 000 *g* for 10 minutes, and the supernatant was collected in a precooled tube.

### Co-Immunoprecipitation Assay

Following the experimental interventions, chondrocytes underwent analysis using an immunoprecipitation (Co-IP) kit (C600688, BBI Life Science Corporation, Shanghai). Protein A-Agarose was prepared by adding 18 µL per reaction to a spin column, removing the cap, and washing the agarose with 700 µL of PBS, followed by low-speed centrifugation, repeated four times in total. Cells were harvested by centrifugation at 800 r/min for 3 minutes at 4 °C. The supernatant was discarded, and the cell pellet was washed twice with pre-chilled PBS, each time spinning at 800 r/min for 3 minutes and ensuring complete drainage of PBS to retain the cell pellets. To 100 mg of cell pellet, 1 mL of 1X Lysis buffer was added, vortexed, and homogenized using a glass homogenizer for 30 strokes or sonicated for 30-second pulses interspersed with 1-minute intervals, repeated thrice. The lysate was then centrifuged at 12 000 r/min for 5 minutes to collect the supernatant containing cell lysate. In a new microcentrifuge tube, 0.7 mL of cell lysate, 7 µL of PMSF, and 1 µg of purified antibody were combined and incubated overnight at 4 °C on a flat platform. Subsequently, the mixture was added to the washed Protein A beads in the spin column and incubated overnight at 4 °C. Each column was placed into supplied 2 mL microcentrifuge tubes and centrifuged at 12 000 *g* for 30 seconds at 4 °C. The beads were washed six times with 700 µL of 1X IP buffer, followed by one wash with 0.1X IP buffer, each wash involving centrifugation at 12 000 *g* for 30 seconds. To the beads, 50 µL of 1X Loading buffer was added and gently mixed (avoiding vortexing). The spin column was securely capped, and the samples were heated at 95 °C for 5 minutes, with excess water around the cap blotted with tissue paper or filter paper. After opening the column cap, it was reinserted into a fresh microcentrifuge tube and centrifuged at 12 000 *g* for 30 seconds. The eluted immunoprecipitate was subsequently subjected to SDS-PAGE analysis, and the proteins were detected using Western blot techniques as previously described.

### Mitochondrial membrane potential detection

Mitochondrial membrane potential was evaluated using fluorescence microscopy with a JC-1 staining kit (Beyotime). Chondrocytes were treated with a 5 μmol/L JC-1 staining solution for 20 minutes at 37 °C in the dark and observed using a Zeiss Axiovert 40CFL microscope. The level of mitochondrial membrane potential (*ΔΨm*) was quantified by measuring the ratio of red at 590 nm to green fluorescence at 525 nm intensities.

### Mitochondrial superoxide detection

Chondrocytes were cultured on coverslips in culture media and subjected to the respective treatments. Mitochondrial superoxide production was assessed using MitoSOX Red (Thermo Fisher Scientific). Cells were washed with PBS, followed by incubation with 1 µmol/L MitoSOX Red for 30 minutes at 37 °C. Following incubation, the coverslips were mounted on glass slides. Fluorescence was visualized under a microscope, and images were analyzed using image analysis software.

### NADH level measurement

The cellular NADH content was determined using a Coenzyme I NAD (H) content test kit (Jiancheng, Nanjing, China). Cells were collected and centrifuged to remove the supernatant. The volume of alkaline extraction solution used was adjusted according to cell density, typically 500–1 000 µL per 10^4^ cells, with 1 mL recommended for 5 × 10^6^ cells. Samples underwent ultrasonic disruption at 200 W or 20% power for 1 minute in an ice bath, pulsing 2 seconds on and 1 second off. Boiling with a secure lid for 5 minutes minimized evaporation. After cooling in an ice bath, debris was pelleted by centrifugation at 10 000 *g* for 10 minutes at 4 °C. The resulting supernatant was transferred to a fresh tube and neutralized by adding an equivalent volume of acidic solution. Additional centrifugation at 10 000 *g* for 10 minutes at 4 °C clarified the supernatant, which was stored on ice for subsequent analysis. Thorough mixing was crucial to dissolve any purple-black precipitate, with residual white sediment removed by centrifugation at 20 000 *g* for 5 minutes at room temperature. Absorbance was measured at 570 nm using a 1 cm path length cuvette, with distilled water as the reference blank. The spectrophotometer was preheated for at least 30 minutes before use. Reagents were prepared in appropriate working dilutions for the simultaneous analysis of multiple samples, protected from light to avoid photometric interference. NADH concentration was determined from absorbance values using a validated formula.

### ATP level

Intracellular ATP levels were quantified using an ATP detection kit (Beyotime). Chondrocytes were lysed with ATP lysis buffer, followed by centrifugation to separate the supernatant. The supernatant was then mixed with the ATP detection reagent and the absorbance of the resulting mixture was measured using a Varioskan LUX multifunctional plate reader (Thermo Fisher Scientific). ATP concentrations were normalized relative to the total protein content.

### Seahorse XF analysis

The Seahorse Extracellular Flux XFe24 Analyzer (Agilent, USA) was employed to investigate extracellular acidification rates (ECARs). Cells were seeded in hippocampal XF-24 plates at a density of 1 × 10^5^ cells per well. Before measurement, cells underwent preconditioning under hypoxic conditions. ECARs were assessed by sequentially administering glucose, oligomycin, and 2-deoxyglucose to the cells, allowing for the evaluation of glycolysis and glycolytic capacity through subsequent calculations and analyses.

### Measurements of mitochondrial complex activity

The mitochondrial complex I-V activity assay kit (Elabscience, China) was used to evaluate mitochondrial complex activity. Cells were collected and carefully mixed with the reagent, then sonicated on ice at 200 W (5 seconds on, 10 seconds off, for 15 cycles). After centrifugation at 10 000 *g* for 3 minutes at a low temperature, the supernatant was extracted for further analysis. The absorbance was measured using a multifunction plate reader (Thermo Fisher Scientific).

### Genetic knockout model

The genetic knockout model involved acquiring global *Sirt3* knockout (*Sirt3*^−/−^) mice from GemPharmatech Co., Ltd. (Nanjing, China) which were subsequently bred with global *Pink1* knockout (*Pink1*^−/−^) mice (Nanjing, China) to generate *Sirt3*^−/−^*Pink1*^−/−^ double-knockout (DKO) mice. Age-matched group consisted of wild-type (WT) siblings from the C57BL/6 J lineage. Genetic characterization of these mice utilized tail-derived DNA following protocols established by the Jackson Laboratory. The animals were housed under controlled conditions with consistent temperature with 12 h light/dark cycles and humidity and maintained on a standard diet.

### Animal model

The animal model involved medial meniscal (DMM) surgery performed on seven-week-old male C57BL/6 J mice, conducted by protocols approved by the Soochow University Ethics Committee (SUDA20240307A07) and adhering to National Institutes of Health guidelines. Mice were obtained from the Soochow University Laboratory Animal Center and anesthetized with sodium pentobarbital before surgery. Following anesthesia, the knee joint area was prepared by shaving, disinfecting, and making an incision from the distal patellar to the proximal tibial region. The joint capsule was then opened, the patellar tendon retracted, and the medial meniscal ligament was exposed and transected. The Sham group underwent a similar incision procedure without transection of the medial meniscotibial ligament. Animals received penicillin to prevent infection and were housed with access to food and water in a designated area for mobility. One week post-surgery, mice, excluding those in the sham operation group (*n* = 5), were randomly assigned to four groups (*n* = 5) for respective interventions and group allocation. Joint samples were collected at 4, 8, and 12 weeks before euthanasia for the first group, whereas all remaining groups were assessed at the 8-week time point.

### Human OA specimens

Tibial plateau samples were collected from patients with OA who were scheduled for total knee arthroplasty, following their provision of informed consent. Specimens from OA cartilage were classified as relatively healthy cartilage or severely damaged cartilage. Exclusion criteria included the presence of malignant tumors, diabetes mellitus, or other significant chronic conditions. Ethics approval was obtained from The First Affiliated Hospital of Soochow University (Approval No. 2022-520).

### Intra-articular injection treatment

Intra-articular injection treatment involved inducing OA in mice through DMM surgery following established protocols. Subsequently, mice were categorized into different groups, consisting of six mice each group. Post-recovery from DMM surgery, saline, AAV-NC, AAV-*Pink1*, AAV-*Sirt3*, siNC, or si*Pink1* was injected into the joint cavity (3 days post-DMM, 1 × 10^11^ v.g./mL, 5 μL per joint). The animals were euthanized at week 8 for subsequent examination and analysis. The procedure for grouping other intra-articular cavity injections adheres to the same protocol as described.

### Gait analysis

Gait analysis was conducted except for the initial group, which underwent experiments at 4, 8, or 12 weeks before euthanasia; all subsequent groups were assessed at 8 weeks. Mice were identified by marking their front paws in red and their rear paws in green, with recording paper positioned within a dark enclosure. Subsequently, mice were placed in the dark enclosure and allowed to move freely from one side to the other without external interference. Each mouse underwent three replicate experiments, and three consecutive footprints from the recording paper were selected for statistical analysis following the experiments.

### μCT analysis

Following euthanasia, the knee joints of the mice underwent tissue fixation. Subsequently, high-resolution micro-computed tomography (μCT) was conducted using a Skyscan 1176 system from Kontich, Belgium, operating at a resolution of 9 μm with settings of 50 kV and 200 μA. Two-dimensional image reconstruction was conducted using NRecon v1.6 workstation and CTAn v1.13.8.1 software. A three-dimensional model was reconstructed using Mimics Research software. Regions of interest (ROI) were defined as 30 consecutive layers of subchondral bone on the medial side of the tibia. Statistical analysis of the reconstructed data evaluated parameters including bone volume fraction (BV/TV, %), trabecular thickness (Tb.Th, mm), and trabecular separation (Tb.Sp, mm).

### Transmission electron microscopy

Isolated mice chondrocytes were fixed for 2 h using Glutaraldehyde (G916053, Macklin, China) at room temperature. The samples were then embedded in epoxy resin and dehydrated using a series of ethanol concentrations. The sections were subsequently visualized using a transmission electron microscope (HT7700, Tokyo, Japan).

### Histological assessment

Knee specimens were fixed in 4% paraformaldehyde (Sigma-Aldrich) for 48 hours, and subsequently decalcified in 10% ethylenediaminetetraacetic acid (EDTA, Sigma-Aldrich). Following decalcification, specimens were embedded, sectioned into 6-μm-thick sagittal slices, deparaffinized in xylene, and gradually rehydrated through a series of ethanol solutions. Sections were stained with hematoxylin and eosin (H&E) as well as safranin O-fast green (S.O.). Assessment of cartilage degeneration utilized the Osteoarthritis Research Society International (OARSI) guidelines, and the calculation of the hyaline cartilage (HC) to calcified cartilage (CC) ratio.

### Immunofluorescence and immunohistochemical staining

Cell cultures were seeded into 24-well plates and treated with chondrocytes as described in the intervention above. Subsequently, cells were fixed in 4% paraformaldehyde for 20 min and permeabilized with 0.2% Triton™ X-100 (Sigma-Aldrich) for 15 min. Mouse knee joint slides and cell cultures were then deparaffinized and blocked with QuickBlock™ Blocking Buffer (Beyotime) for 30 minutes. Samples were incubated overnight at 4 °C with specific primary antibodies. After washing with PBS, cells were treated with Fluor488-conjugated or CY3-conjugated secondary Antibodies at room temperature for 1 h. Nuclei were counterstained with DAPI (Thermo Fisher Scientific) for 1 minute and resulting immunofluorescence images were visualized using a fluorescence microscope. Mitochondrial autophagy staining was performed using the Mitophagy Detection Kit (Dojindo Molecular Technologies, Japan) following the manufacturer’s instructions. Cells treated with Ad-GFP-LC3B (Beyotime) underwent co-staining for lysosomes using Lyso Tracker Red (1:20 000, Beyotime) after a 30-minute incubation at 37 °C. Subsequently, cells were fixed with 3% glutaraldehyde, as previously described. Mitochondria were co-stained with PINK1 using Mito Tracker Red (1:3 000, Beyotime). After blocking, sections were incubated overnight at 4 °C with respective primary antibodies, followed by procedures similar to those used for cell fluorescence. The Intensity and co-localization of immunofluorescence were quantified using Image J software (National Institutes of Health, Bethesda, MD, USA) for further analysis. Immunohistochemical staining of slices was performed using M&R HRP/DAB Detection IHC Kit (Vazyme).

### Colocalization analysis

Colocalization analysis of fluorescent signals in chondrocytes was performed using a plugin to compute Pearson’s Correlation Coefficient (PCC), which is well suited for assessing high-intensity signals within cell. Images were captured from multiple coverslips or well plates under each experimental condition, with each chondrocyte imaged once. PCC ranges from +1 indicating perfect positive correlation to −1 indicating perfect negative correlation, providing a quantitative measure of the linear relationship between signal intensities in two channels. Due to its robustness against background noise, PCC serves as an effective tool for colocalization analysis.

### Mitochondrial length analysis

Mitochondrial length analysis commenced following the steps of immunofluorescence. Cells were incubated overnight with an anti-TOMM20 antibody, washed with PBS, and incubated with a CY3-conjugated secondary antibody for 1 hour. Following this, cells were co-stained with DAPI for 1 minute. Microscopic observations were conducted and images were captured accordingly. At least 10 cells were randomly selected from each field of view to measure mitochondrial length, with the mean length calculated subsequently. The quantitative analysis involved determining the mean mitochondrial lengths obtained from 5 randomly selected fields of view. Mitochondrial branch length analysis utilized Fiji software through binary and skeletonization processes.

### High intelligent and sensitive structured illumination microscopy (HIS-SIM)

Super-resolution imaging of cartilage cells under specific interventions was performed using the HIS-SIM system (CSR Biotech, Guangzhou, China). Following standard procedures, GFP was excited at 488 nm with a 525/20 nm filter, and mCherry at 561 nm with a 610/20 nm filter. Images were captured with a 100x/1.5 NA oil immersion objective from Olympus. Analysis of the 3D-SIM data was conducted using Imaris software, after processing with the HIS-SIM technology, which included Wiener deconvolution of the reconstructed images.

### Phosphoproteomics sequencing and bioinformatic analysis

Phosphoproteomics sequencing and bioinformatic analysis involved several systematic steps. Initially, proteins underwent enzymatic digestion to produce peptides. Phosphopeptides were selectively enriched from this peptide mixture using techniques such as metal affinity chromatography, immobilized metal affinity chromatography (IMAC), or titanium dioxide (TiO2) chromatography. This enrichment step enhances the concentration of phosphopeptides while reducing sample complexity. Next, the enriched phosphopeptides were separated using liquid chromatography (LC) and subsequently introduced into a mass spectrometer (MS) for analysis. Tandem mass spectrometry (MS/MS) was employed to fragment the peptides, generating a spectrum of fragment ions. These MS/MS spectra were then compared against protein sequence databases using specialized software tools to identify phosphorylated peptides. The sequencing service was facilitated by Wekemo Tech Group Co., Ltd., ensuring rigorous analytical procedures. Identified differentially expressed proteins underwent further analysis through Gene Ontology (GO) and Kyoto Encyclopedia of Genes and Genomes (KEGG) pathway assessments using the online platform “OMICSHARE” (www.omicshare.com).

### Statistical analysis

Statistical analysis was conducted using SPSS 13.0 (SPSS Inc., Chicago, IL, USA) and results are presented as mean ± standard deviation (SD). One-way analysis of variance (ANOVA) was employed for multiple comparisons while two-tailed non-paired Student’s *t*-tests were used for specific pairwise comparisons. Statistical significance was set at *P* < 0.05.

## Supplementary information

Supplementary Data
