# Supplementary material for: SIRT3-PINK1-PKM2 axis prevents osteoarthritis via mitochondrial renewal and metabolic switch
Source: Bone Res. 2025 Mar 14;13:36. doi: 10.1038/s41413-025-00413-4 (PMC11909255; doi:10.1038/s41413-025-00413-4)
Supplement: Supplementary file 1 — Supplementary Data [file 41413_2025_413_MOESM1_ESM.docx]

***SIRT3-PINK1-PKM2 axis prevents osteoarthritis via*** ***mitochondrial renewal and metabolic switch***

Yaoge Deng^1,2,#^, Mingzhuang Hou^1,2,#^, Yubin Wu^1,2,#^, Yang Liu^1,2^, Xiaowei Xia^1,2^, Chenqi Yu^1,2^, Jianfeng Yu^1,2^, Huilin Yang^1,2,*^, Yijian Zhang^1,2,*^, Xuesong Zhu^1,2,*^

^1^Department of Orthopaedics, The First Affiliated Hospital of Soochow University, Soochow University, Suzhou 215006, China

^2^Orthopaedic Institute, Medical College, Soochow University, Suzhou 215000, China

^#^These authors contributed equally to this work.

^*^Correspondence: suzhouspine@163.com (H.Y.); zhangyijian@suda.edu.cn (Y.Z.); zhuxs@suda.edu.cn (X.Z.)

**Supplementary Figures**

**
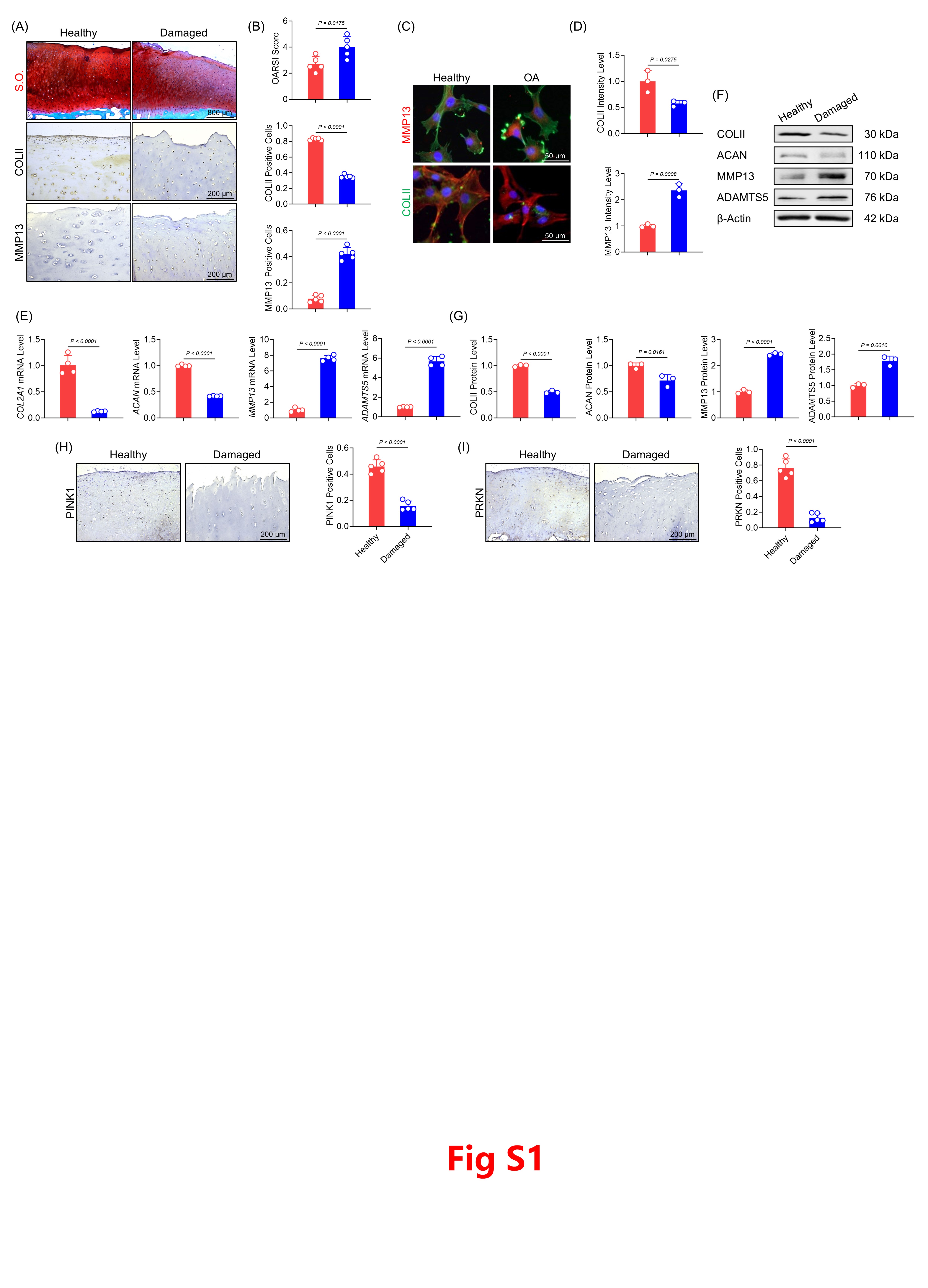
**

**Figure S1.** (A-B) Representative images of Safranin O (S.O.) staining, immunohistochemical (IHC) staining for COLII or MMP13, and quantification analysis of the OARSI scale, as well as the proportion of COLII- or MMP13-positive chondrocytes relative to the total chondrocyte count in smooth and damaged cartilage from osteoarthritis (OA) patients (n = 5). (C-D) Quantification of COLII or MMP13 immunofluorescence intensities in chondrocytes from smooth and damaged cartilage of OA patients (n = 3). (E) mRNA expression analysis of *Col2a1*, *Acan*, *Mmp13*, and *Adamts5* using polymerase chain reaction (n = 4). (F-G) Protein levels of COLII, ACAN, MMP13 and ADAMTS5 in chondrocytes from smooth and damaged cartilage of OA patients (n = 3). (H-I) Quantification of IHC staining for PINK1- or PRKN-positive chondrocytes in smooth and damaged cartilage from osteoarthritis (OA) patients (n = 5). The values represent mean ± SD. Statistically significant differences are indicated by *P* < 0.05 between the indicated groups.


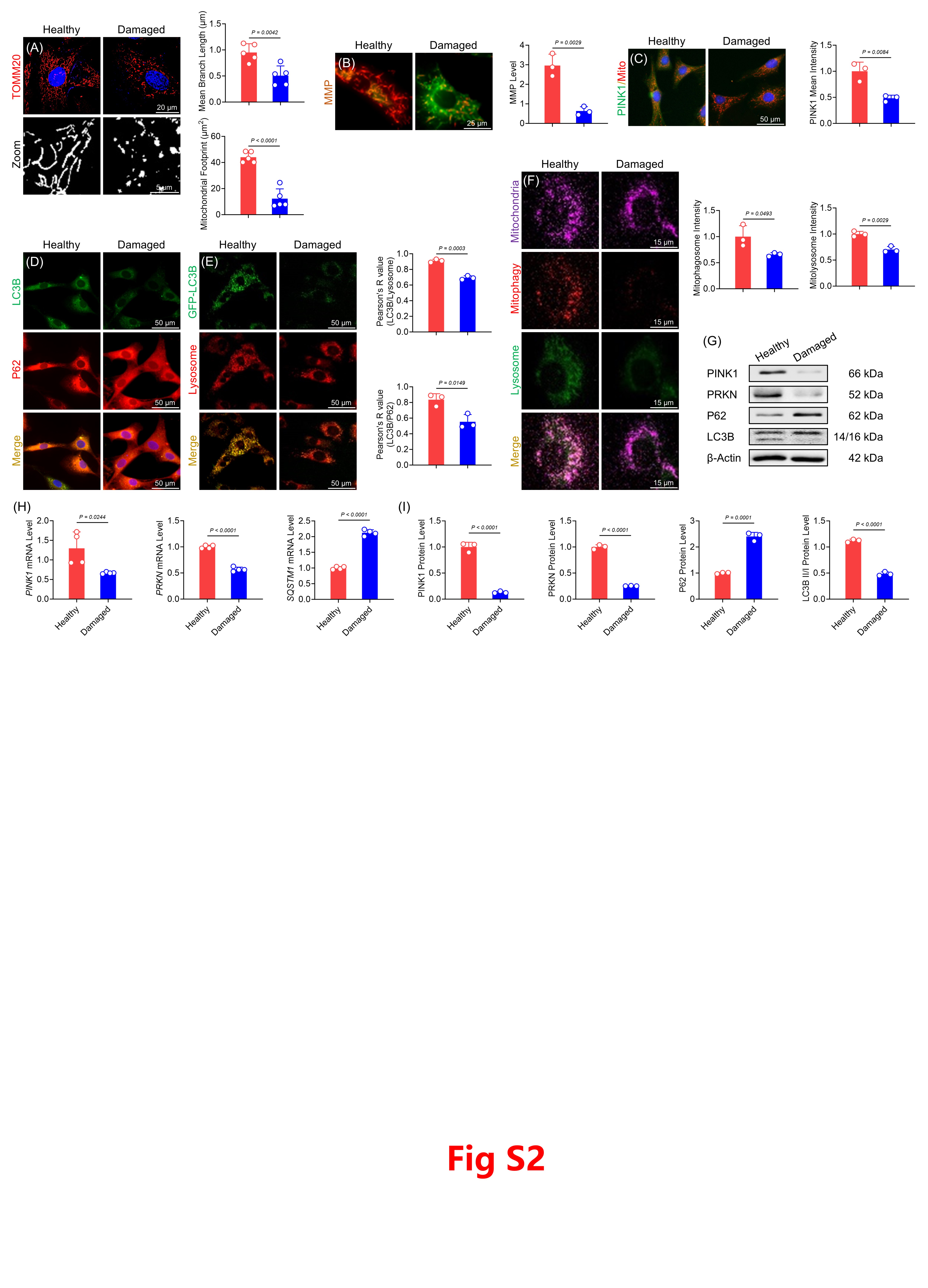


**Figure S2.** (A) Mitochondrial length and footprint measurement via translocase of outer mitochondrial membrane 20 (TOMM20) immunofluorescence in human articular chondrocytes (n = 5). (B) Mitochondrial membrane potential (MMP) assessed using JC-1 staining and fluorescence microscopy in the same group (n = 3). (C) Co-staining of mitochondria and PINK1 using immunofluorescence (n = 3). (D-E) Evaluation of P62 or lysosome colocalization with LC3B in human articular chondrocytes (n = 3). (F) Assessments of mitophagy fluorescence intensity in the same group (n = 3). (G-I) Analysis of PINK1-PRKN axis-related protein mRNA (n = 4) and protein levels (n = 3) in chondrocytes from smooth and damaged cartilage of OA patients. The values represent mean ± SD. Statistically significant differences are indicated by *P* < 0.05 between the indicated groups.


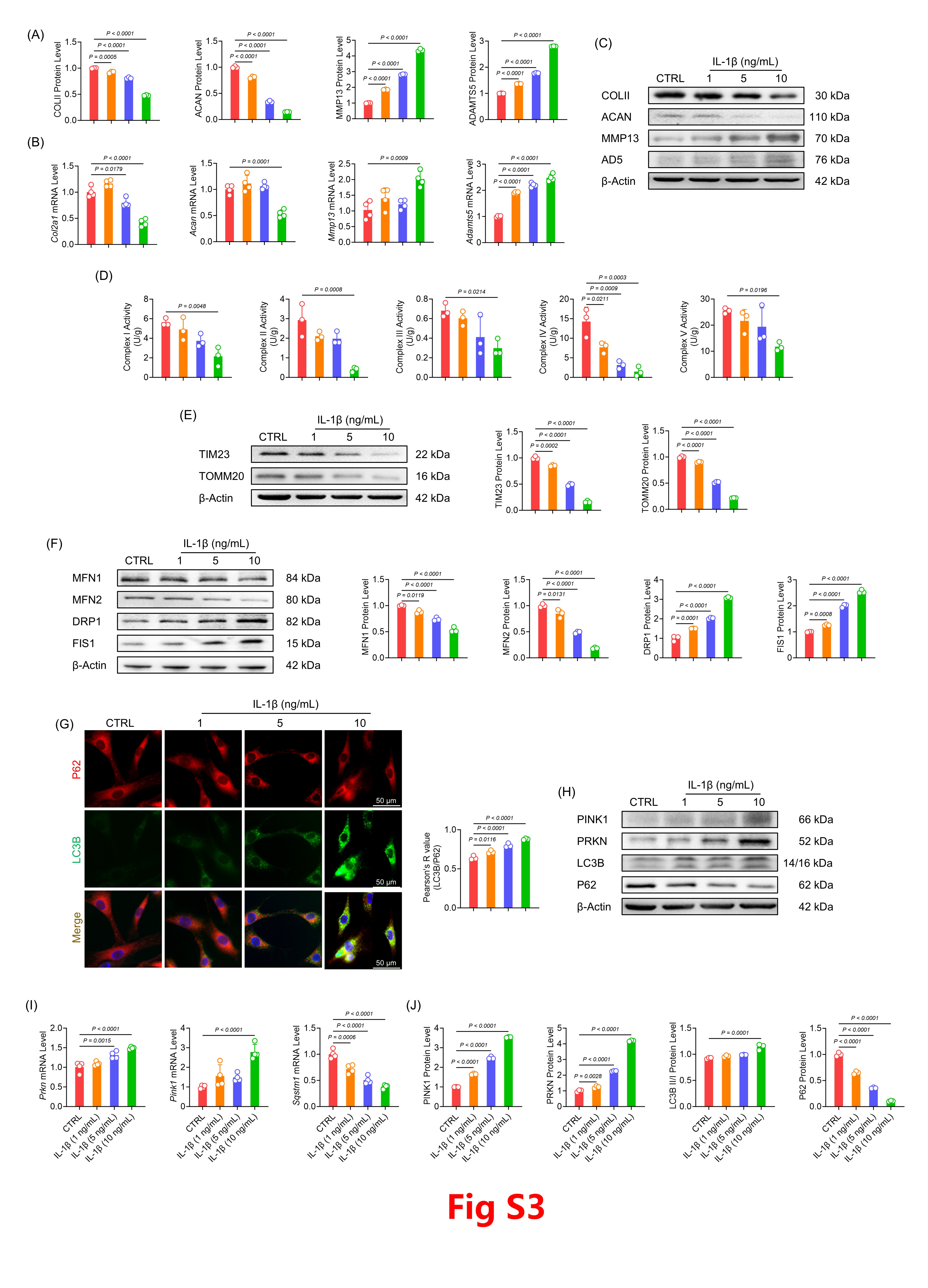


**Figure S3.** (A-C) Analysis of cartilage matrix-related protein mRNA (n = 4) and protein levels (n = 3). (D) Measurements of mitochondrial respiratory chain complex I-V activity (n = 3). (E) Western blot analysis of TIM23 and TOMM20 protein levels (n = 3). (F) Western blot analysis of MFN1, MFN2, DRP1, and FIS1 protein levels (n = 3). (G) Evaluation of P62 colocalization with LC3B in chondrocytes following treatments with IL-1β with concentration gradient (n = 3). (H-J) Analysis of PINK1-PRKN axis-related protein mRNA (n = 4) and protein levels (n = 3) in the same group. The values represent mean ± SD. Statistically significant differences are indicated by *P* < 0.05 between the indicated groups.


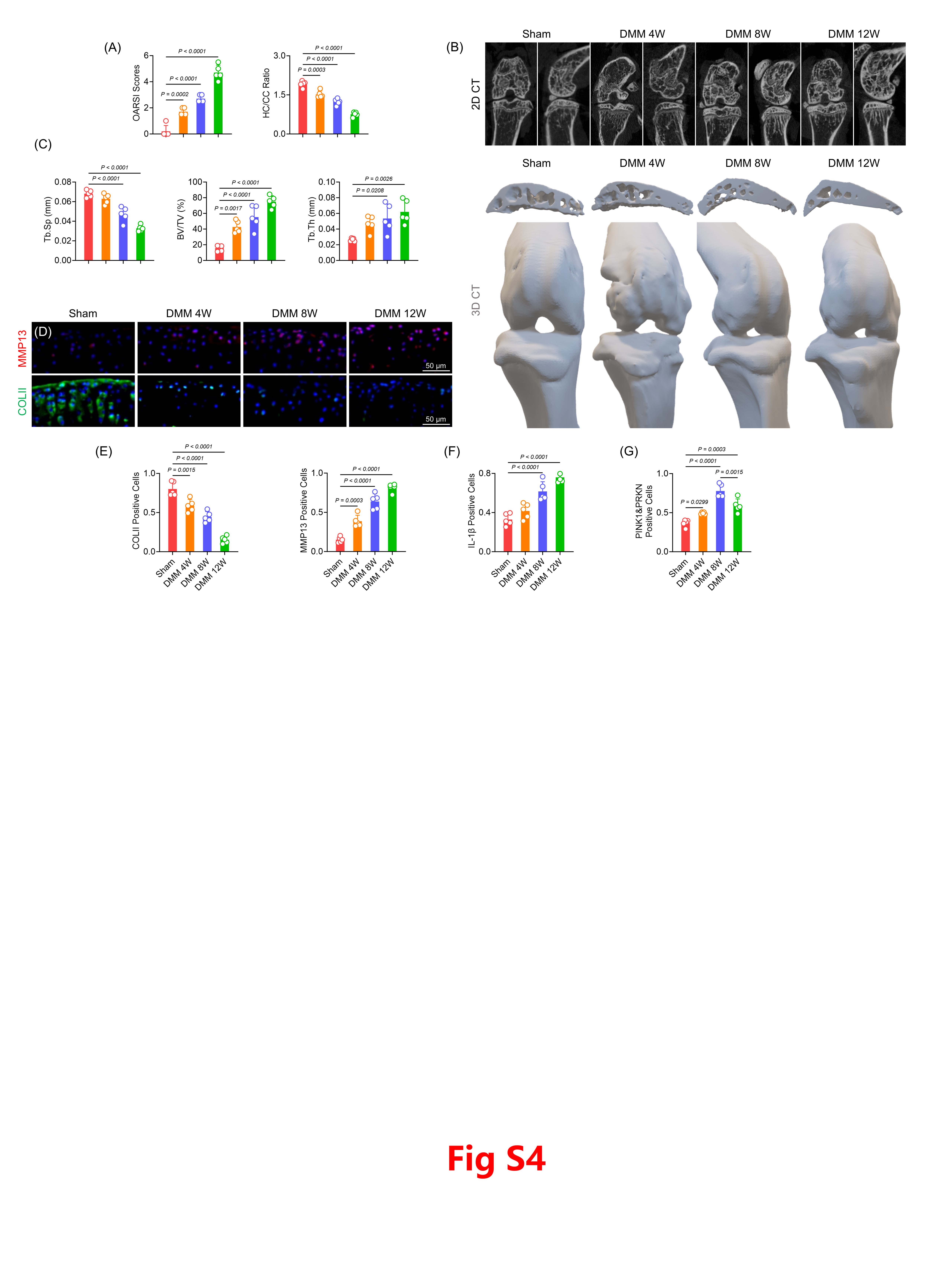


**Figure S4.** (A) Quantification of the OARSI score and the HC/CC ratio in Sham and destabilization of the medial meniscus (DMM) mice (n = 5). (B-C) μ-CT imaging assessments of subchondral bone in corresponding mouse groups (n = 5). (D-E) *In vivo* quantification of COLII- or MMP13-positive chondrocytes via immunofluorescence in corresponding mouse groups (n = 5). (F) *In vivo* quantification of IL-1β-positive chondrocytes via immunofluorescence (n = 5). (G) *In vivo* quantification of PINK1- and PRKN-positive chondrocytes (n = 5). The values represent mean ± SD. Statistically significant differences are indicated by *P* < 0.05 between the indicated groups.


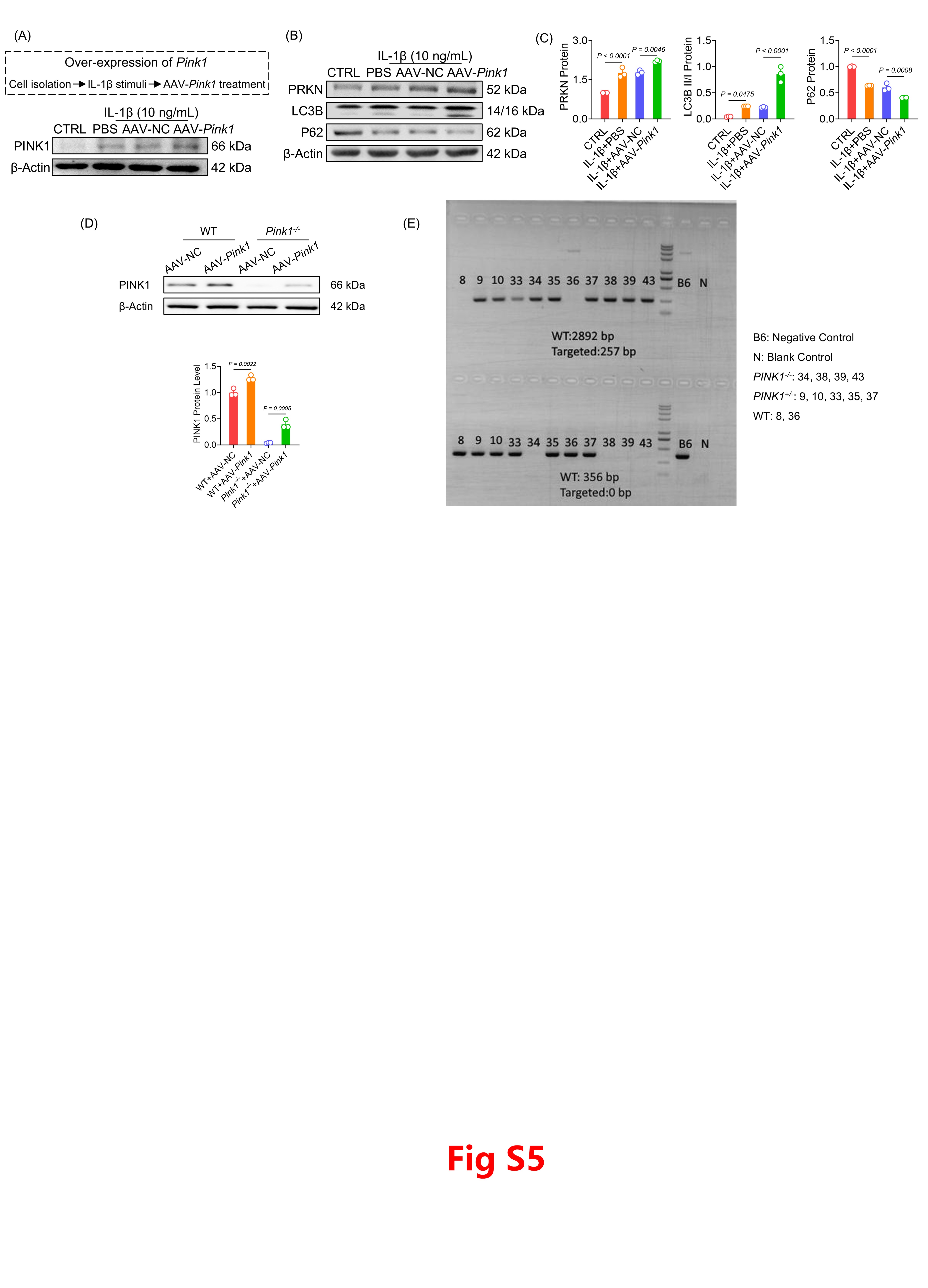


**Figure S5.** (A) Adeno-associated virus (AAV)-mediated overexpression of *Pink1* elevates PINK1 protein levels. (B-C) Upregulation of PRKN-P62-LC3B mitophagy pathway proteins following *Pink1* overexpression (n = 3). (D) Compared the protein levels of PINK1 in chondrocytes between wild-type and *Pink1^–/–^* mice after the overexpression of *Pink1* (n = 3). (E) Identification of *Pink1^–/–^* mice genotype using sodium dodecyl sulfate-polyacrylamide gel electrophoresis (SDS-PAGE). The values represent mean ± SD. Statistically significant differences are indicated by *P* < 0.05 between the indicated groups.


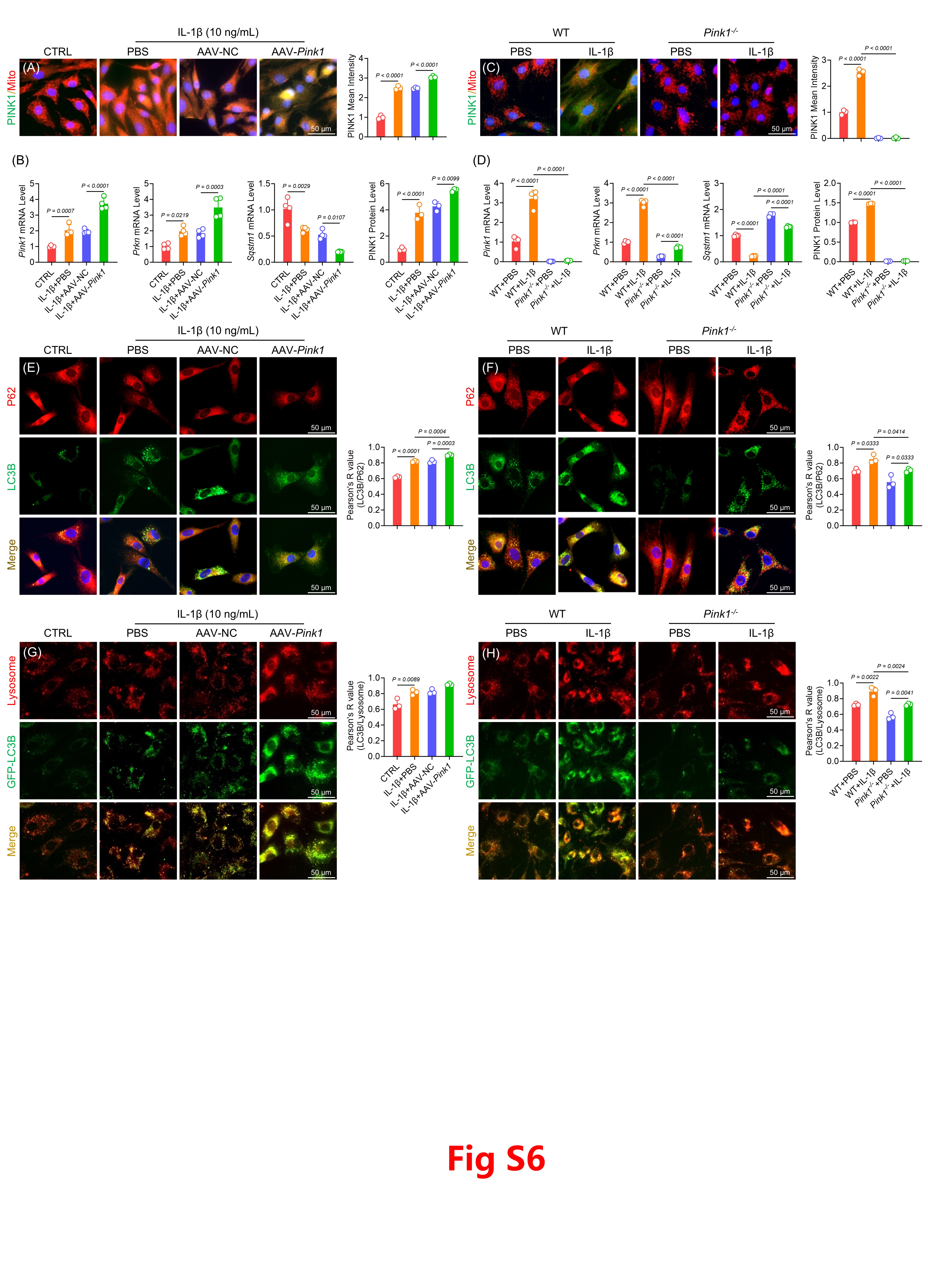


**Figure S6.** (A and C) Analyses of the colocalization of LC3B and lysosomes upon overexpression and knockout of *Pink1*, respectively (n = 3). (B and D) Analyses of PINK1-PRKN axis-related protein mRNA (n = 4) and protein levels (n = 3) in chondrocytes following overexpression and knockout of *Pink1*. (E-H) Evaluation of P62 or lysosome colocalization with LC3B in chondrocytes following overexpression and knockout of *Pink1* (n = 3). The values represent mean ± SD. Statistically significant differences are indicated by *P* < 0.05 between the indicated groups.


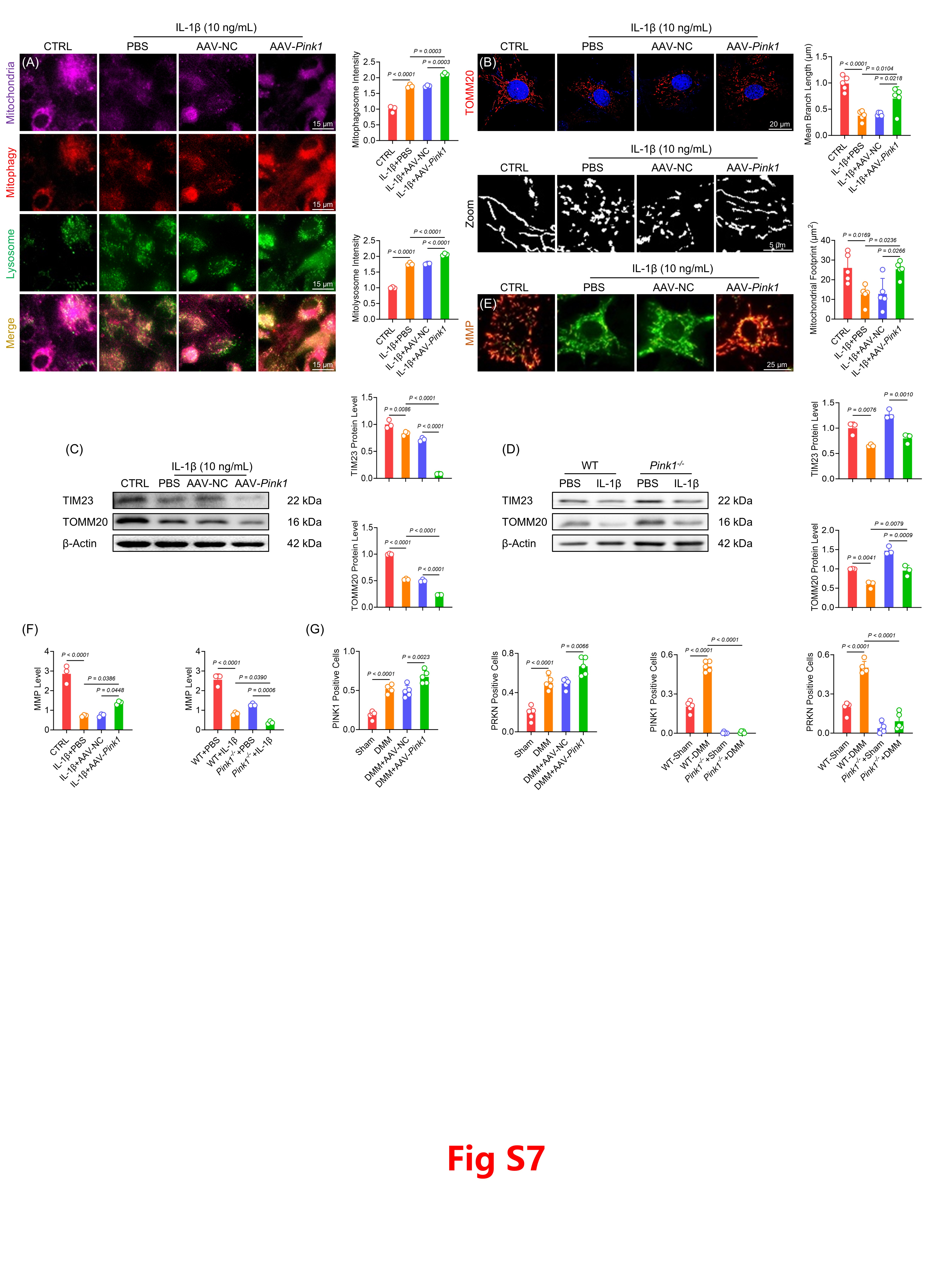


**Figure S7.** (A) Assessments of mitophagy fluorescence intensity following overexpression of *Pink1* (n = 3). (B) Assessments of mitochondrial length and footprint via TOMM20 immunofluorescence (n = 5). (C-D) Protein levels of TIM23 and TOMM20 following overexpression and knockout of *Pink1* (n = 3). (E-F) Detection of MMP changes using JC-1 staining subsequent to *Pink1* overexpression and deficiency (n = 3). (G) *In vivo* quantification of positive cells for PINK1-PRKN axis-related proteins following *Pink1* overexpression and deficiency (n = 5). The values represent mean ± SD. Statistically significant differences are indicated by *P* < 0.05 between the indicated groups.


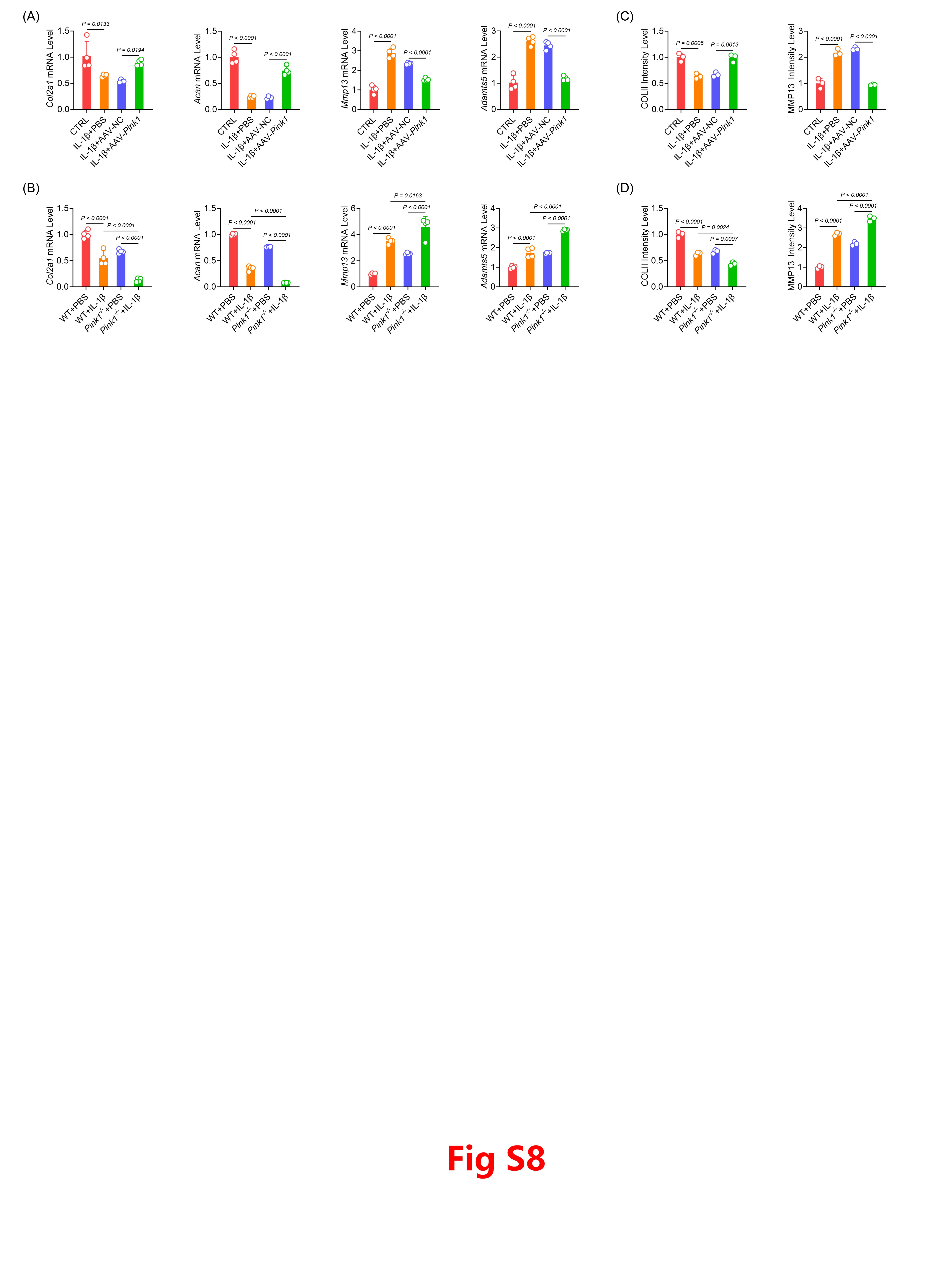


**Figure S8.** (A-B) Gene expression analysis of *Col2a1*, *Acan*, *Mmp13*, and *Adamts5* following overexpression and knockout of *Pink1* (n = 4). (C-D) Quantification of immunofluorescence intensity for COLII and MMP13 following overexpression and knockout of *Pink1* (n = 3). The values represent mean ± SD. Statistically significant differences are indicated by *P* < 0.05 between the indicated groups.


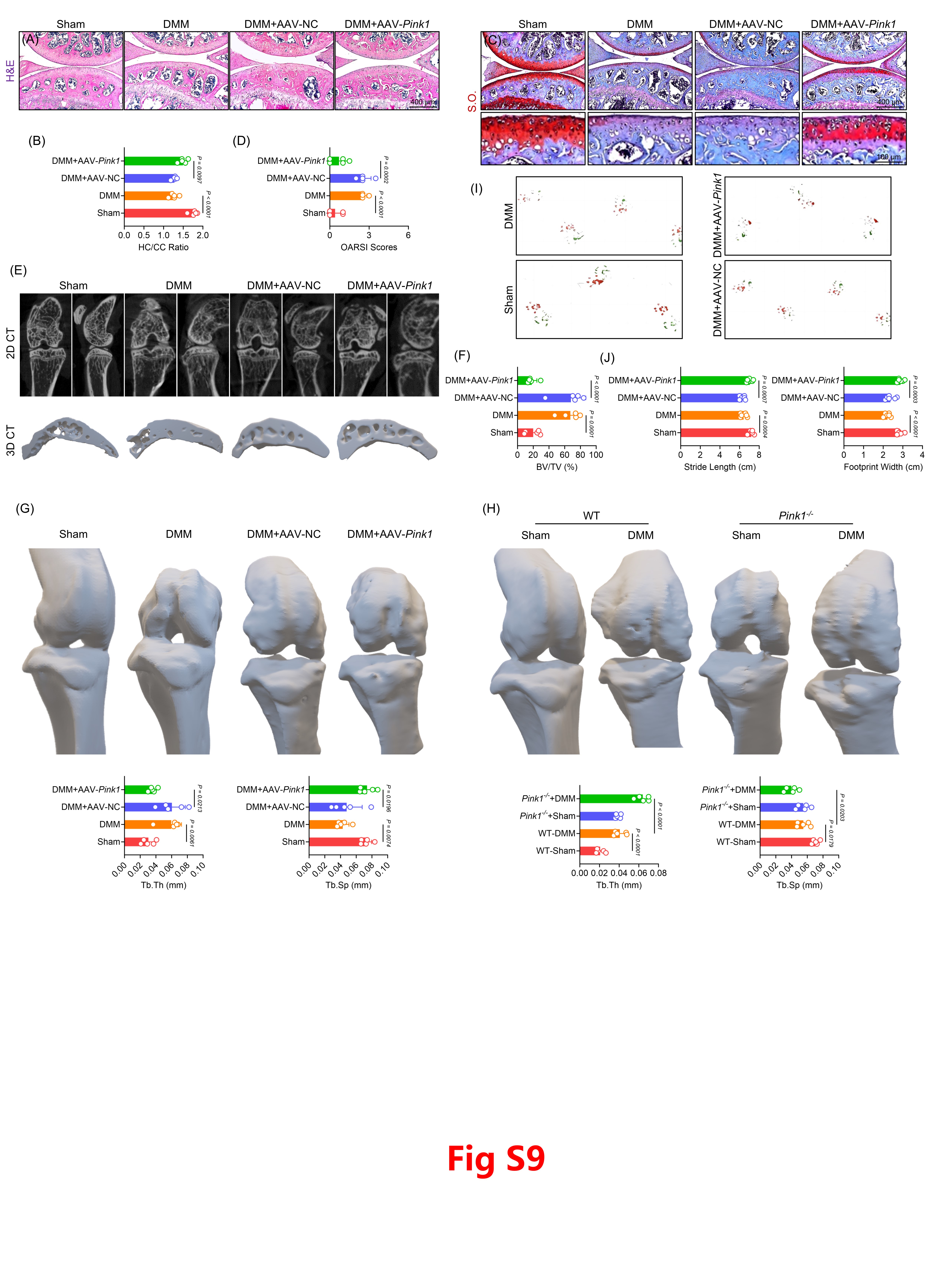


**Figure S9.** (A-D) H&E and S.O. staining following intra-articular administration of AAV-*Pink1* analyzed using OARSI scores and the HC/CC ratio (n = 5). (E–G) μ-CT imaging analyses of mice following intra-articular administration of AAV-*Pink1* (n = 5). (H) μ-CT imaging analyses of mice following knockout of *Pink1* (n = 5). (I-J) Gait analyses of mice following intra-articular administration of AAV-*Pink1* (n = 6). The values represent mean ± SD. Statistically significant differences are indicated by *P* < 0.05 between the indicated groups.


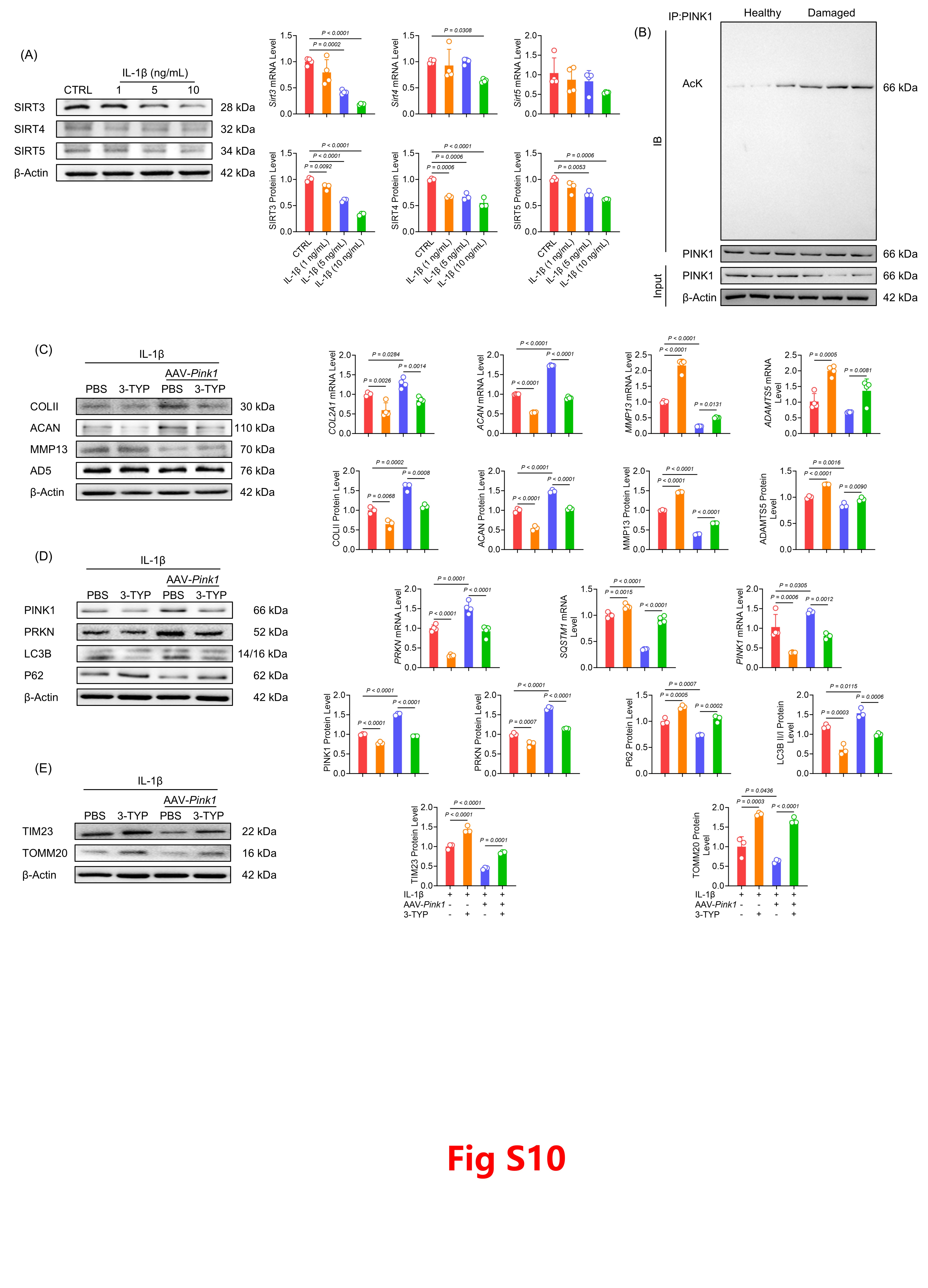


**Figure S10.** (A) Protein levels (n = 3) and mRNA expression (n = 4) of SIRT3, SIRT4, and SIRT5 following treatments with IL-1β with concentration gradient. (B) Detection of total protein content and acetylation level of PINK1 in human healthy or damaged chondrocytes using Co-IP assay. (C-D) Protein levels (n = 3) and mRNA expression (n = 4) of cartilage matrix-related and PINK1-PRKN axis-related proteins in human articular chondrocytes under the treatment of IL-1β, AAV-*Pink1*, and 3-TYP. (E) Protein levels of TIM23 and TOMM20 in the same group (n = 3). The values represent mean ± SD. Statistically significant differences are indicated by *P* < 0.05 between the indicated groups.


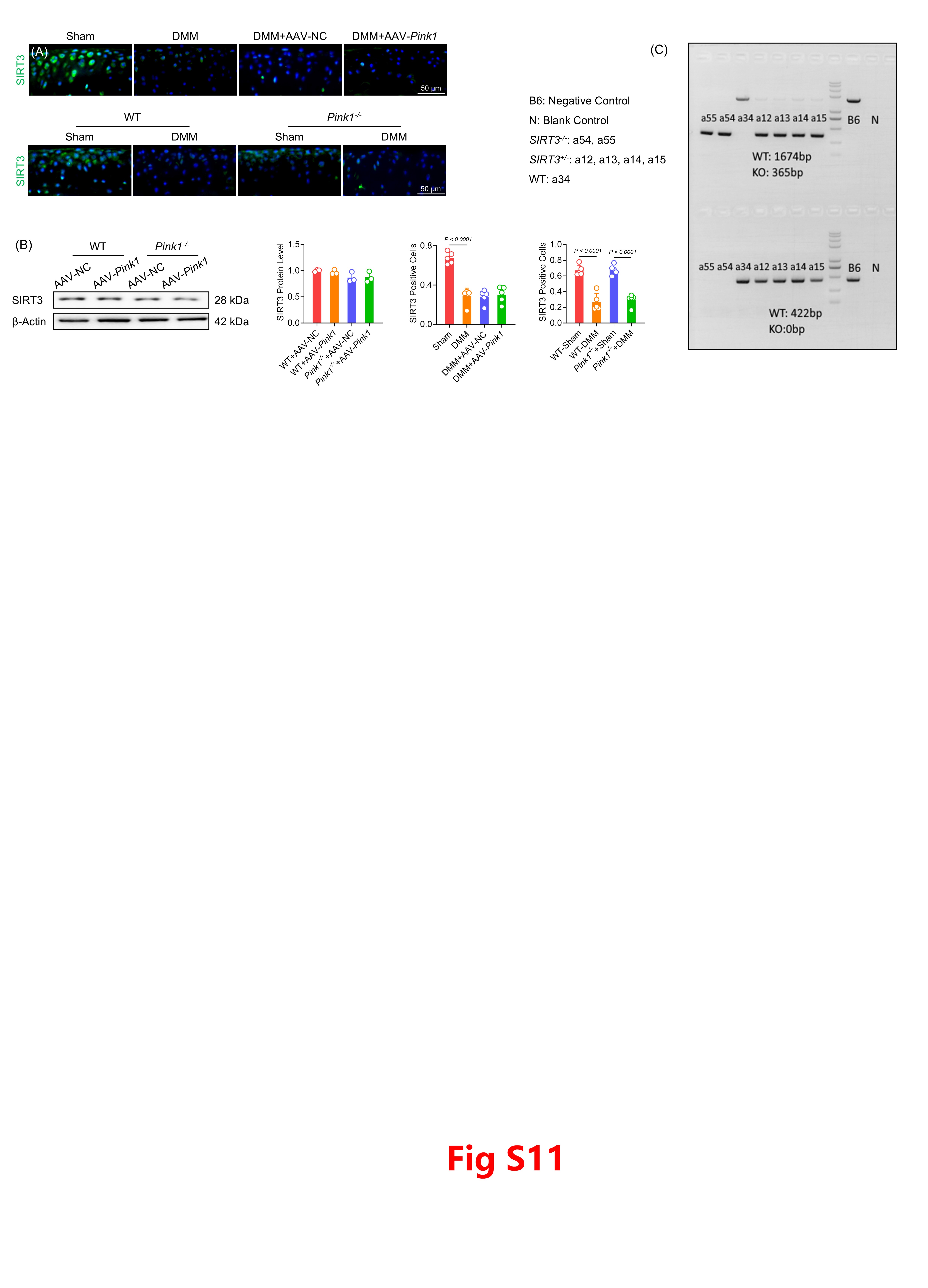


**Figure S11.** (A) *In vivo* quantification of SIRT3-positive chondrocytes following *Pink1* overexpression and knockout (n = 5). (B) Compared the protein levels of SIRT3 in chondrocytes between wild-type and *Pink1^–/–^* mice after the overexpression of *Pink1* (n = 3). (C) Genotypic identification of *Sirt3^–/–^* mice utilizing SDS-PAGE. The values represent mean ± SD. Statistically significant differences are indicated by *P* < 0.05 between the indicated groups.


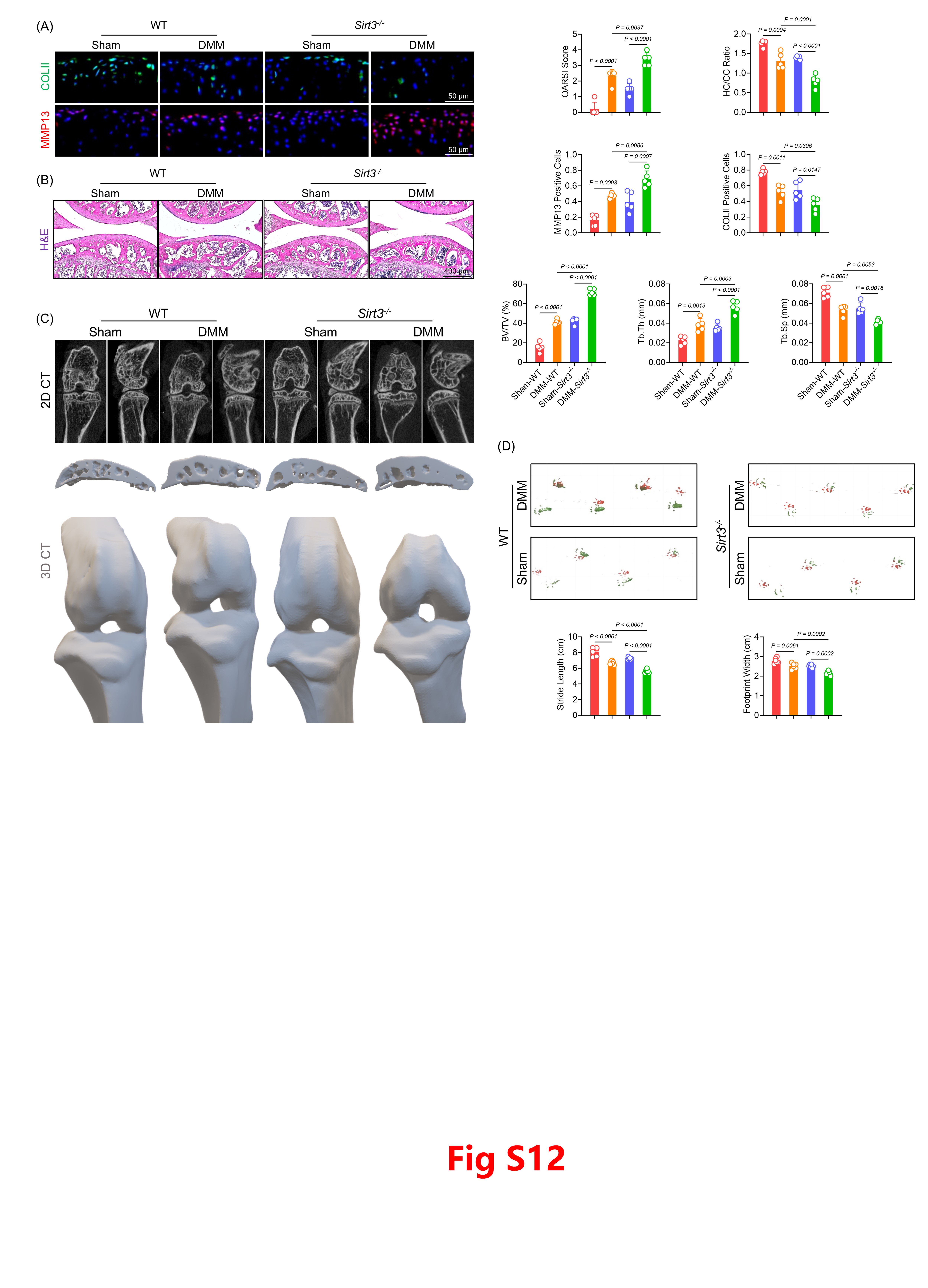


**Figure S12.** (A) *In vivo* representative images of COLII- or MMP13-positive chondrocytes via immunofluorescence in tissue sections from 8-week post-surgery Sham and DMM mice across wild-type and *Sirt3^–/–^* groups (n = 5). (B) H&E staining with subsequent OARSI scoring and HC/CC ratio analyses in *Sirt3^–/–^* mice (n = 5). (C) μ-CT imaging assessments of subchondral bone in corresponding mouse groups (n = 5). (D) Gait analysis of wild-type and *Sirt3^–/–^* mice before and after surgery (n = 6). The values represent mean ± SD. Statistically significant differences are indicated by *P* < 0.05 between the indicated groups.


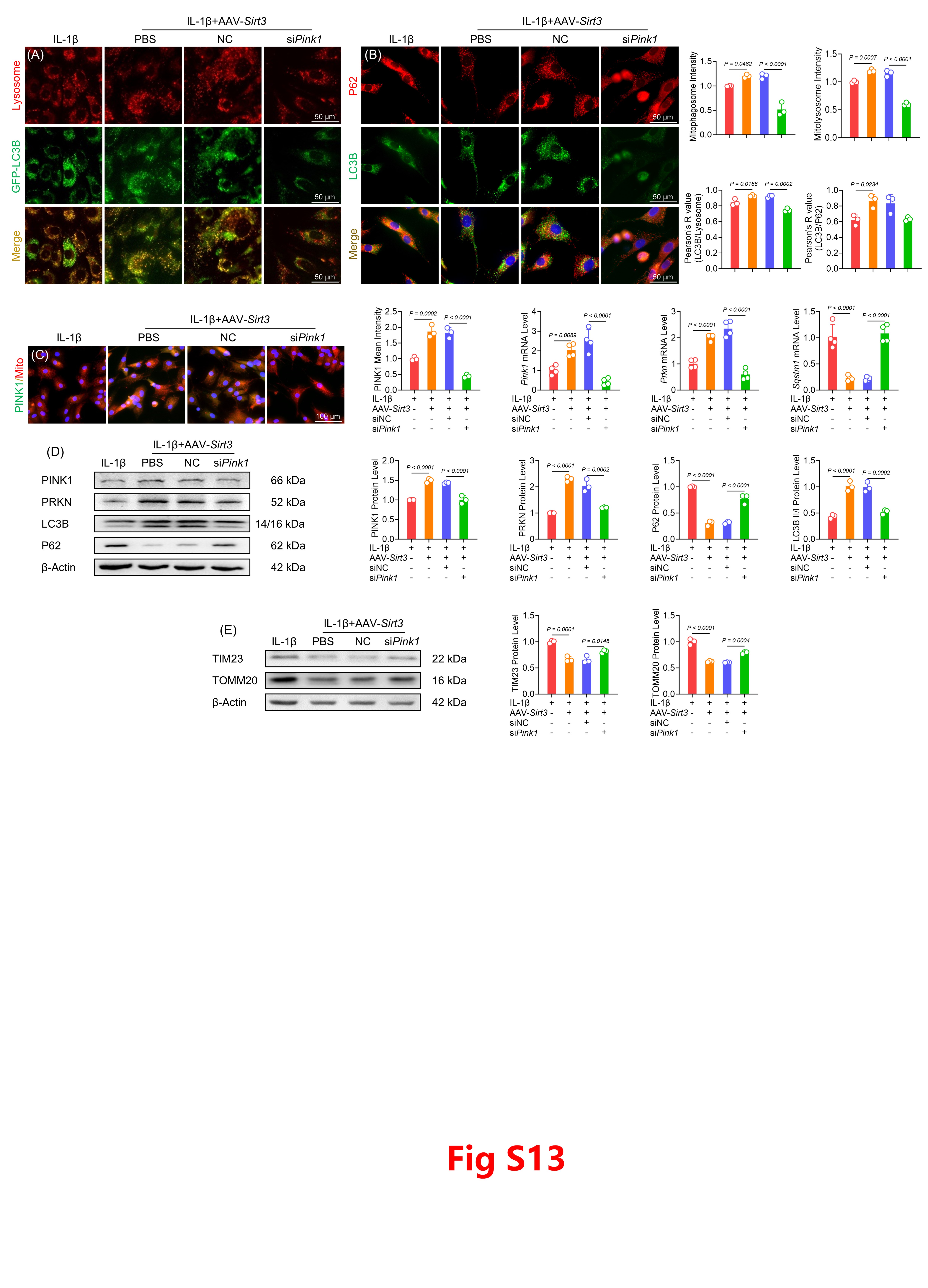


**Figure S13.** (A-B) Evaluation of P62 or lysosome colocalization with LC3B in chondrocytes following *Sirt3* overexpression and subsequent si*Pink1* treatment (n = 3). (C) Co-staining of mitochondria and PINK1 using immunofluorescence and post-treated with si*Pink1* (n = 3). (D) Analyses of protein levels (n = 3) and gene expression (n = 4) for PINK1, PRKN, and P62, as well as LC3B protein levels, after treatment with si*Pink1*. (E) Protein levels of TIM23 and TOMM20 following disruption of the SIRT3-PINK1 interaction (n = 3). The values represent mean ± SD. Statistically significant differences are indicated by *P* < 0.05 between the indicated groups.


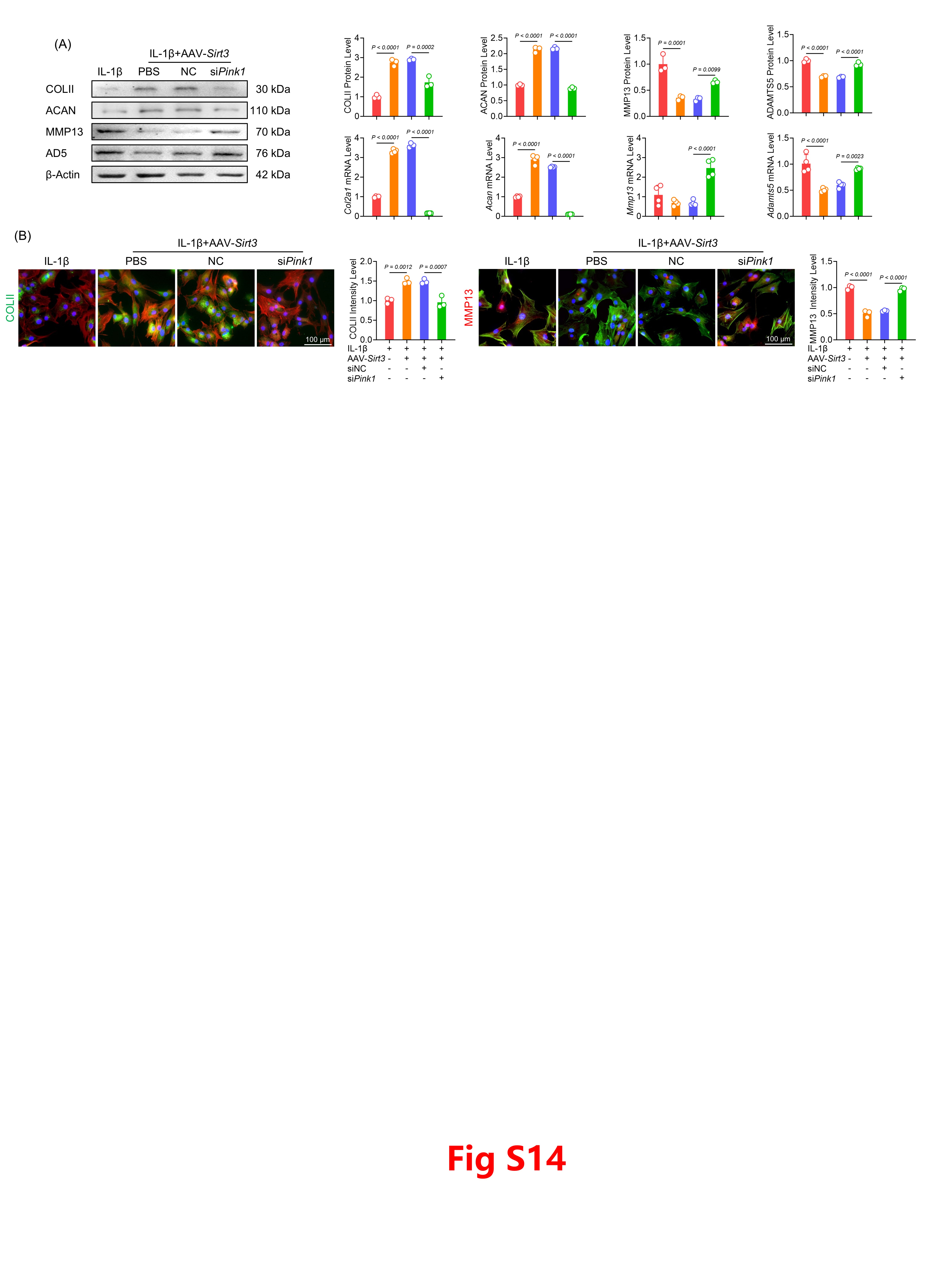


**Figure S14.** (A) Analyses of protein levels (n = 3) and gene expression (n = 4) for cartilage matrix-related proteins following *Sirt3* overexpression and subsequent si*Pink1* treatment. (B) Quantification of immunofluorescence intensity for COLII and MMP13 within the same experimental group (n = 3). The values represent mean ± SD. Statistically significant differences are indicated by *P* < 0.05 between the indicated groups.


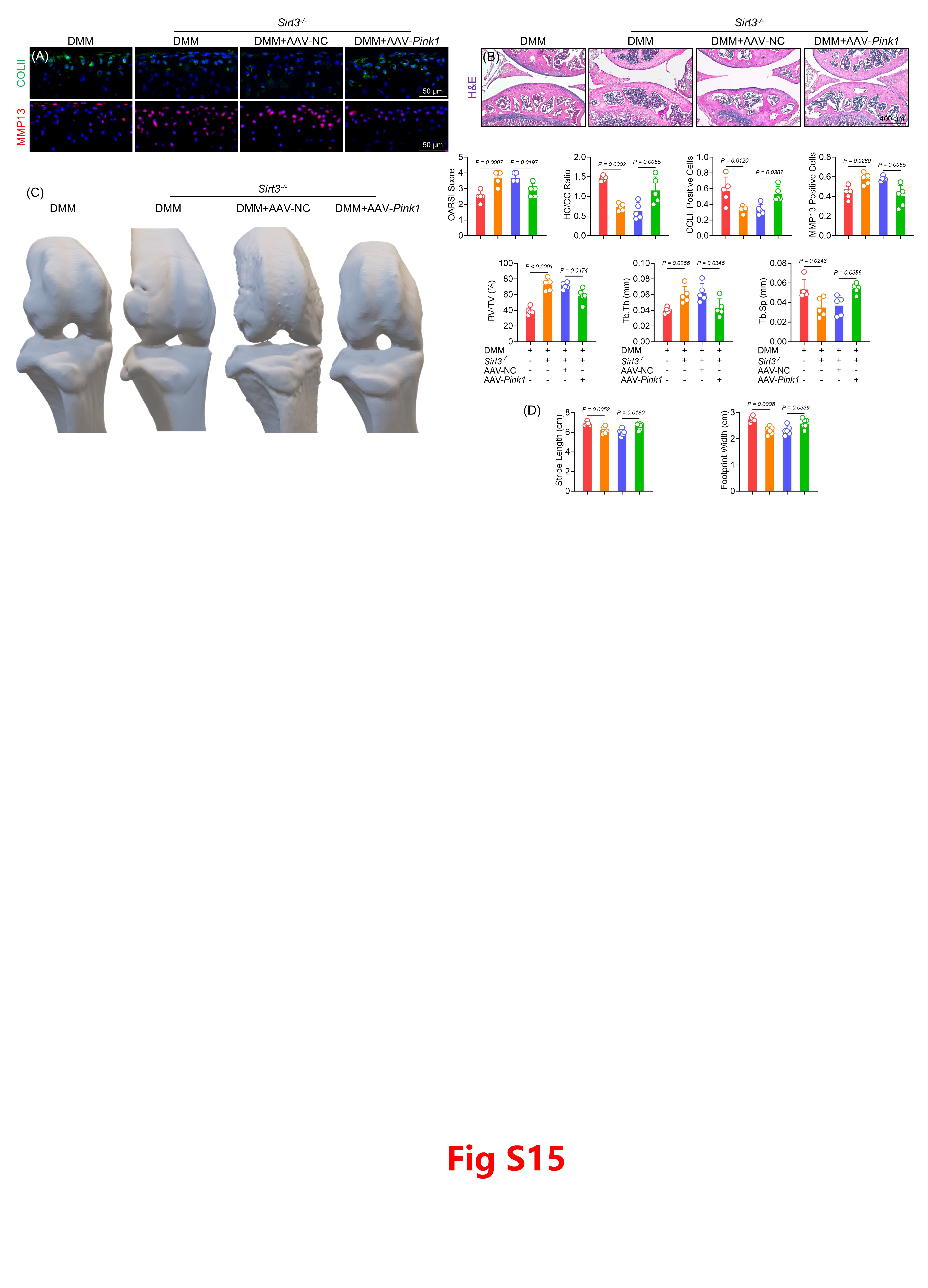


**Figure S15.** (A) *In vivo* Immunofluorescence representative images of COLII- or MMP13-positive chondrocytes for *Sirt3^–/–^* mice before and after overexpression of *Pink1* (n = 5). (B) H&E staining with subsequent OARSI scoring and HC/CC ratio analyses (n = 5). (C) μ-CT imaging assessments of subchondral bone in corresponding mouse groups (n = 5). (D) Gait analysis of mice in corresponding groups (n = 6). The values represent mean ± SD. Statistically significant differences are indicated by *P* < 0.05 between the indicated groups.


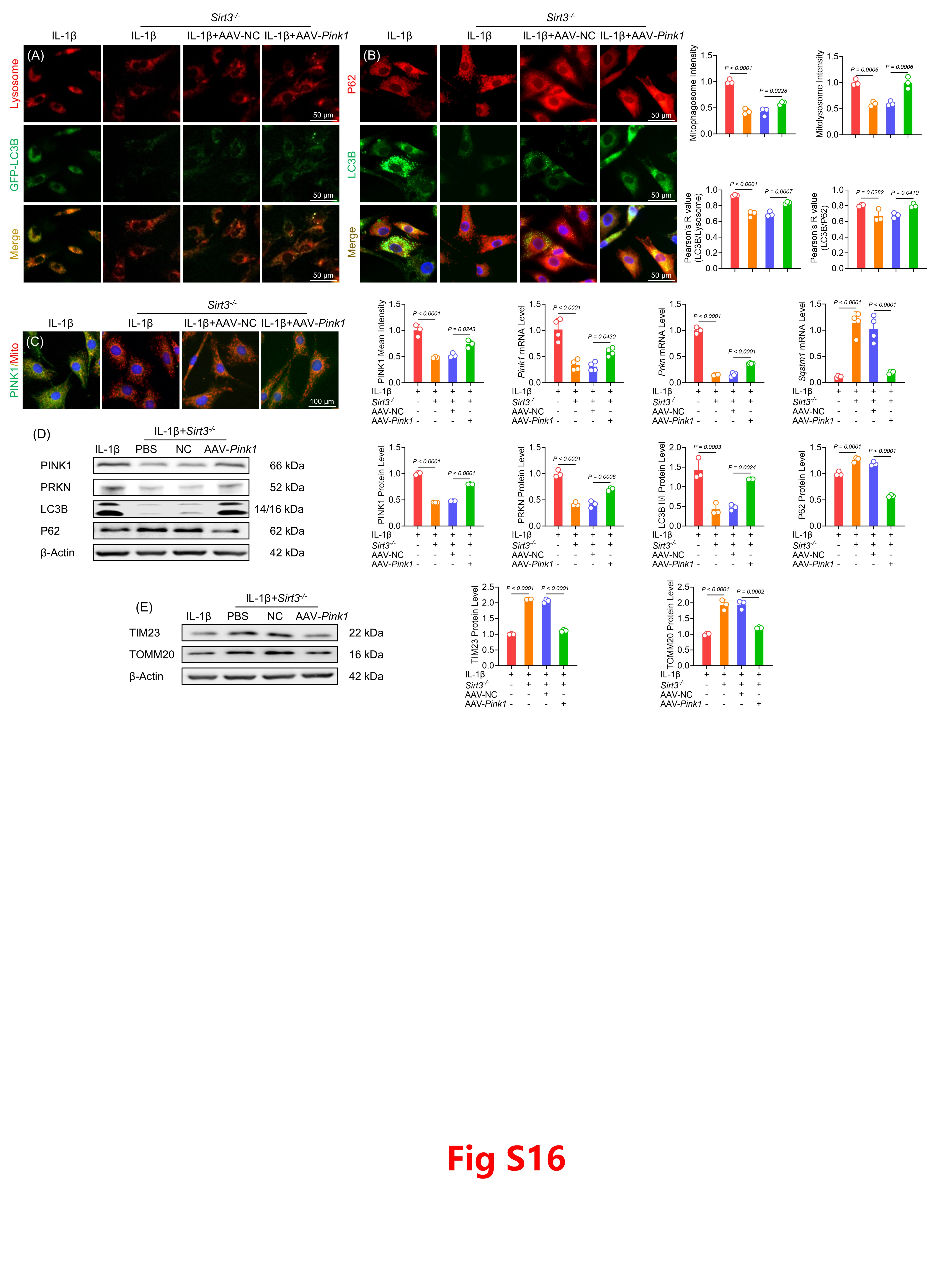


**Figure S16.** (A-B) Evaluation of P62 or lysosome colocalization with LC3B in chondrocytes following *Sirt3* deficiency and subsequent AAV-*Pink1* treatment (n = 3). (C) Co-staining of mitochondria and PINK1 using immunofluorescence following the same treatment (n = 3). (D) Analyses of protein levels (n = 3) and gene expression (n = 4) for PINK1, PRKN, and P62, as well as LC3B protein levels. (E) Protein levels of TIM23 and TOMM20 following *Sirt3* deficiency and subsequent AAV-*Pink1* treatment (n = 3). The values represent mean ± SD. Statistically significant differences are indicated by *P* < 0.05 between the indicated groups.


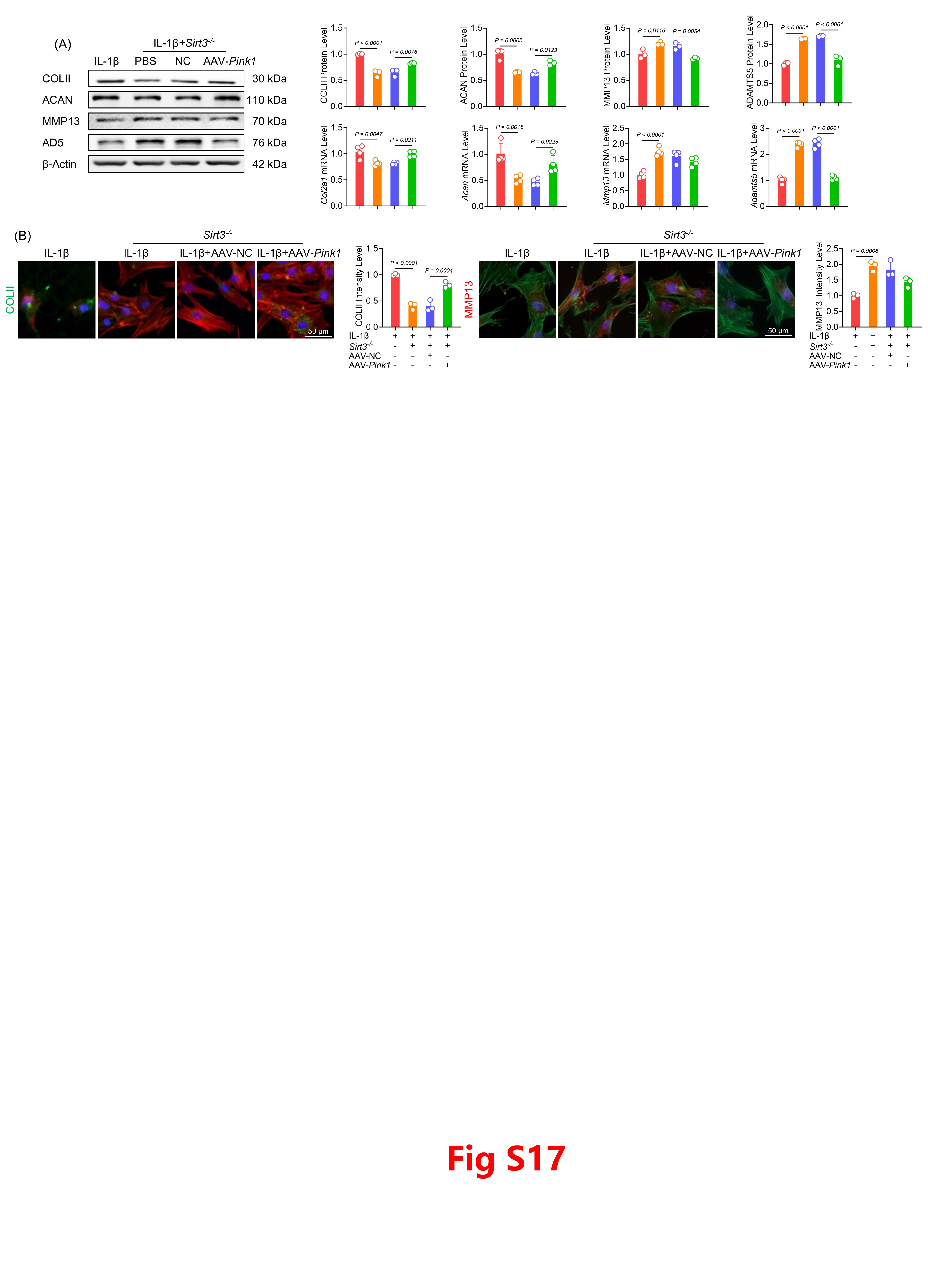


**Figure S17.** (A) Analysis of cartilage matrix-related protein mRNA (n = 4) and protein levels (n = 3) in chondrocytes following *Sirt3* deficiency and subsequent AAV-*Pink1* treatment. (B) Quantification of immunofluorescence intensity for COLII and MMP13 within the same experimental group (n = 3). The values represent mean ± SD. The values represent mean ± SD. Statistically significant differences are indicated by *P* < 0.05 between the indicated groups.


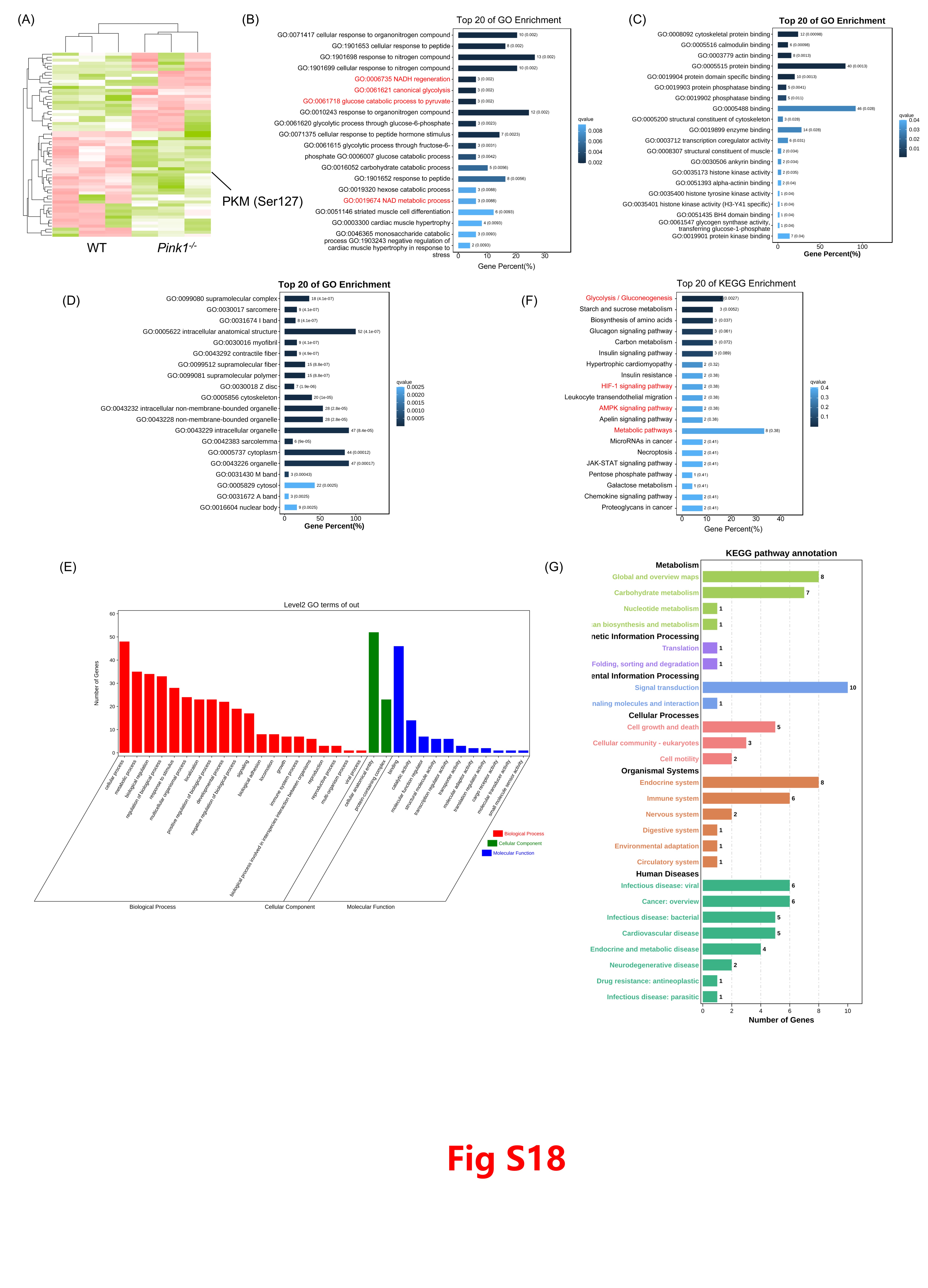


**Figure S18.** (A) Comparative phosphoproteomic profiling of wild-type and *Pink1^–/–^* mice. (B-E) GO enrichment analysis of phosphorylation modifications regulated by PINK1. (F-G) KEGG pathway analysis of PINK1-mediated phosphorylation events.


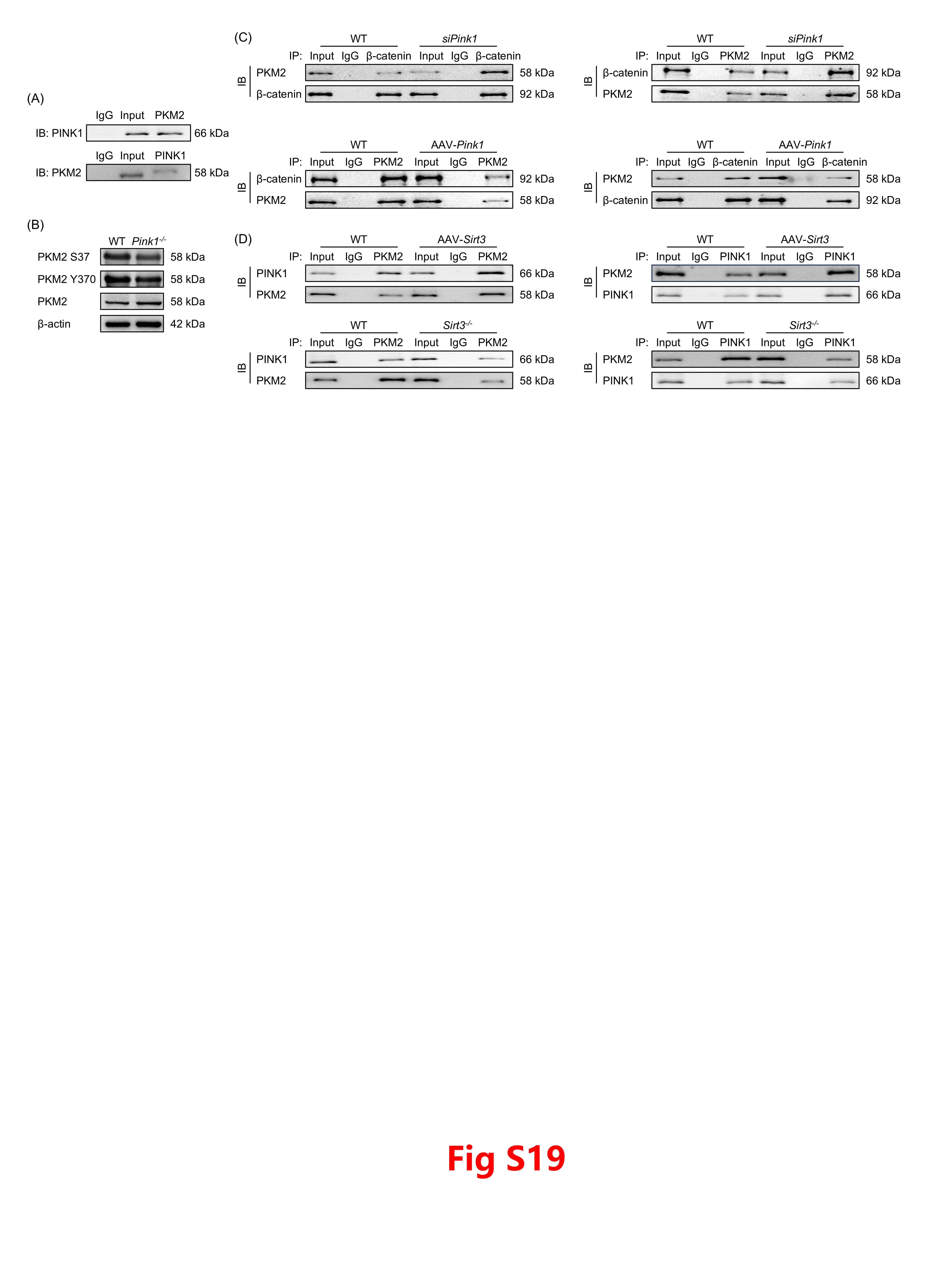


**Figure S19.** (A) Validation of the endogenous PKM2-PINK1 interaction using Co-IP assay. (B) Impact of *Pink1* deletion on PKM2 protein levels and phosphorylation at Tyr370 and Ser37. (C) Immunoblotting analysis revealed that PINK1 deletion led to formation of a PKM2-β-catenin complex, whereas PINK1 overexpression inhibits the interaction between PKM2 and β-catenin. (D) Analyses of the interaction between PINK1 and PKM2 in response to SIRT3 overexpression and knockout via Co-IP assays.


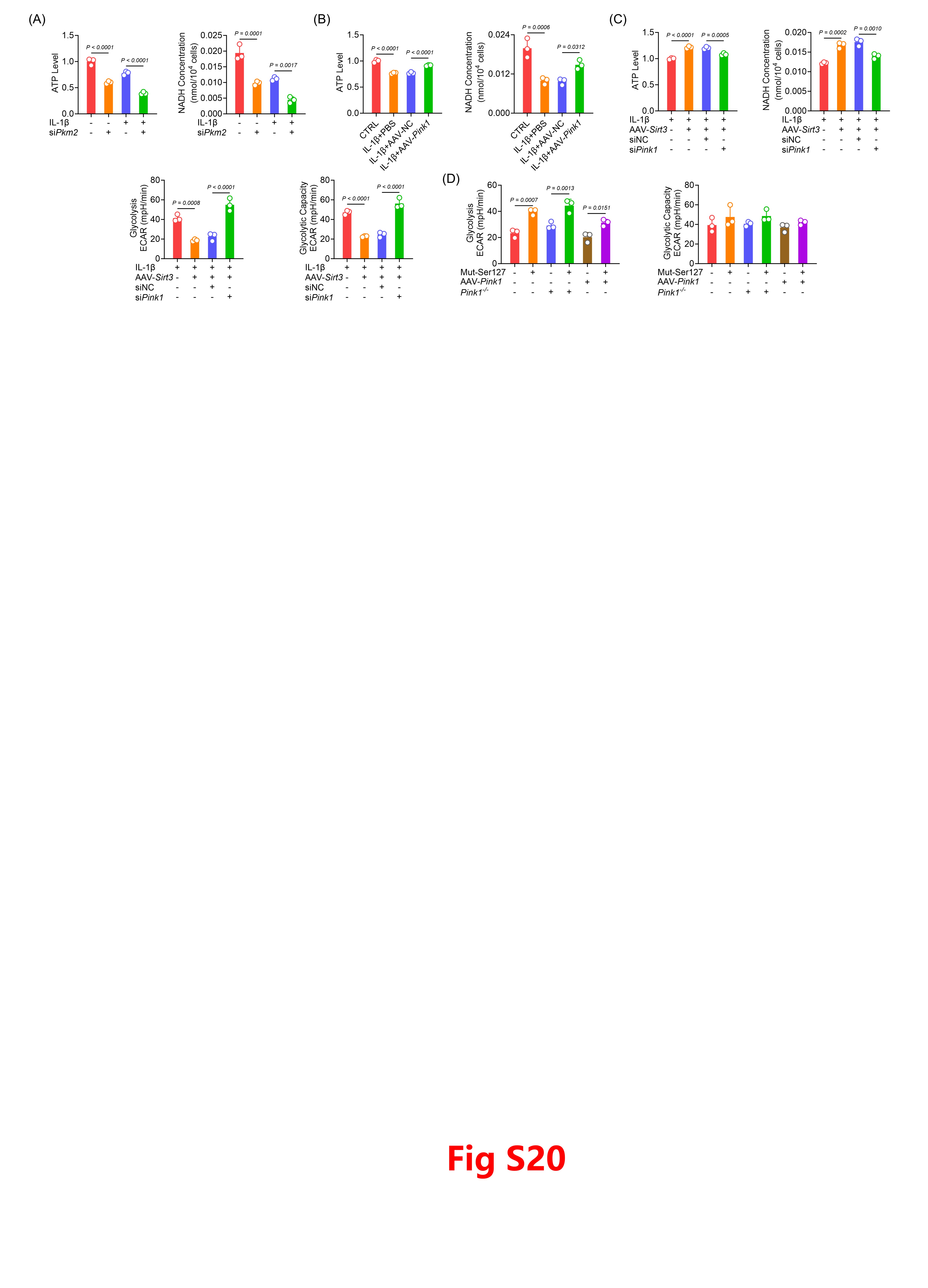


**Figure S20.** (A) Exploration of the effects of IL-1β and si*Pkm2* on ATP and NADH production (n = 3). (B-C) Quantitative of ATP and NADH levels following *Pink1* overexpression or disruption of the SIRT3-PINK1 axis (n = 3). (D) Analysis of glycolysis and glycolytic capacity in corresponding groups (n = 3).


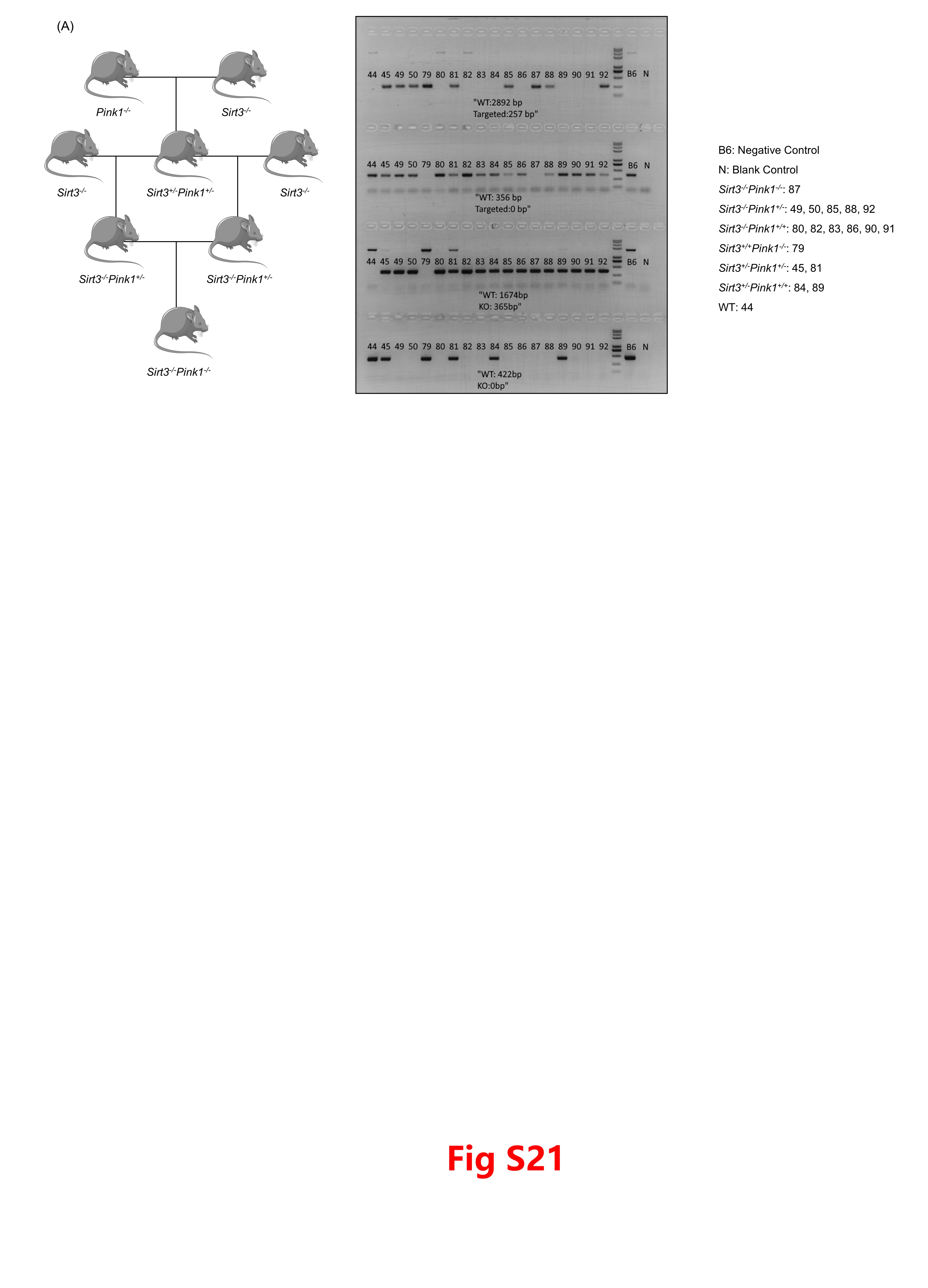


**Figure S21.** (A) The construction process of the double-knockout mouse model and gel diagram for genotype identification.


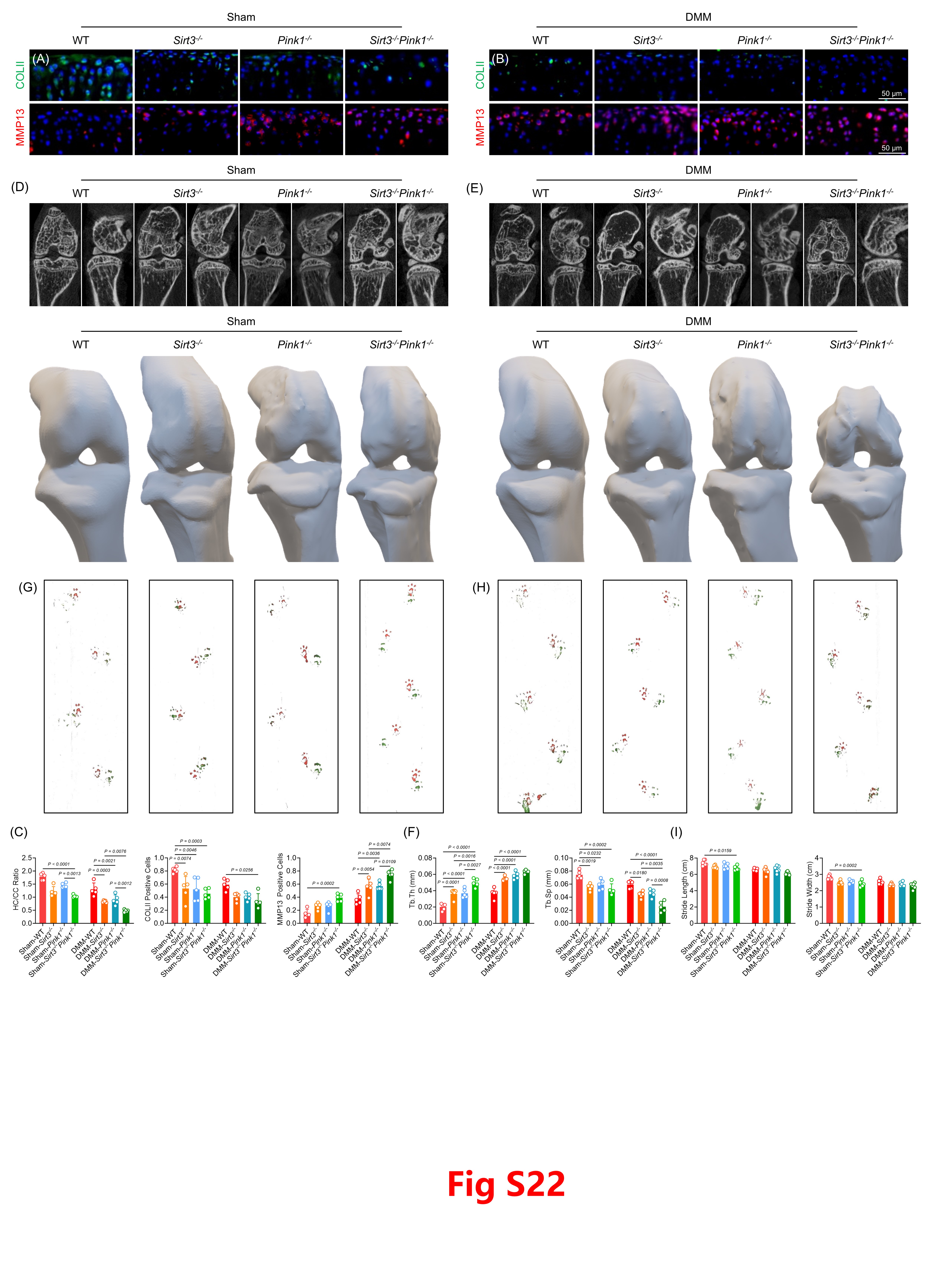


**Figure S22.** (A-C) *In vivo* immunofluorescence analysis of COLII- or MMP13-positive chondrocytes in *Sirt3^–/–^* *Pink1^–/–^* double-knockout mice before and after surgery compared with other genotypes within the same experimental group (n = 5). (D-F) μ-CT imaging assessments of subchondral bone parameters including trabecular thickness (Tb.Th) and trabecular separation (Tb.Sp) in corresponding mouse groups (n = 5). (G-I) Gait analyses of *Sirt3^–/–^* *Pink1^–/–^* double-knockout mice compared with other genotypes within the same experimental group (n = 6). The values represent mean ± SD. Statistically significant differences are indicated by *P* < 0.05 between the indicated groups.

**Supplementary Table 1**. Primers used for quantitative real-time RT-PCR

| **Gene** | **Forward Primer sequence (5'-3')** | **Reverse Primer sequence (3'-5')** |
| --- | --- | --- |
| Mmu-*Gapdh* | AGCCATGTACGTAGCCATCC | CTCTCAGCTGTGGTGGTGAA |
| Mmu-*Col2a1* | CACGCATGAGCCGAAGCTA | GGGTTTCCACGTCTCACCA |
| Mmu-*Acan* | ATTTCCACACGCTACACCCTG | TGGATGGGGTATCTGACTGTC |
| Mmu-*Mmp13* | ACTGAGAGGCTCCGAGAAATG | GAACCCCGCATCTTGGCTT |
| Mmu-*Adamts5* | ACTACGATGCAGCTATCCTGT | GTCCCAACGTCTGCCATTC |
| Mmu-*Pink1* | TTCTTCCGCCAGTCGGTAG | CTGCTTCTCCTCGATCAGCC |
| Mmu-*Prkn* | GAGGTCGATTCTGACACCAGC | CCGGCAAAAATCACACGCAG |
| Mmu-*Sqstm1* | GAGGCACCCCGAAACATGG | ACTTATAGCGAGTTCCCACCA |
| Mmu-*Sirt3* | GCTGCTTCTGCGGCTCTATACAC | CAAAGGTCCCGTGGGCTTCAAC |
| Mmu-*Sirt4* | GCCTCAATTCTCCTCTCACCAACC | GCCGCTGACTCCCTGCTTTG |
| Mmu-*Sirt5* | GTGTACCTCGTGTGGCACTGTTG | GGATTCTGGCGTCTTGAGTCTCTG |

*Mmu: Mus musculus*

| **Gene** | **Forward Primer sequence (5'-3')** | **Reverse Primer sequence (3'-5')** |
| --- | --- | --- |
| Hsa-*Gapdh* | GGAGCGAGATCCCTCCAAAAT | GGCTGTTGTCATACTTCTCATGG |
| Hsa-*Col2a1* | TGGACGCCATGAAGGTTTTCT | TGGGAGCCAGATTGTCATCTC |
| Hsa-*Acan* | ACTCTGGGTTTTCGTGACTCT | ACACTCAGCGAGTTGTCATGG |
| Hsa-*Mmp13* | CCAGACTTCACGATGGCATTG | GGCATCTCCTCCATAATTTGGC |
| Hsa-*Adamts5* | GAACATCGACCAACTCTACTCCG | CAATGCCCACCGAACCATCT |
| Hsa-*Pink1* | GGAGGAGTATCTGATAGGGCAG | AACCCGGTGCTCTTTGTCAC |
| Hsa-*Prkn* | CCCACCTCTGACAAGGAAACA | TCGTGAACAAACTGCCGATCA |
| Hsa-*Sqstm1* | GACTACGACTTGTGTAGCGTC | AGTGTCCGTGTTTCACCTTCC |

*Hsa: Homo sapiens*
